# Measurement of time-varying kinematics of a dolphin in burst accelerating swimming

**DOI:** 10.1371/journal.pone.0210860

**Published:** 2019-01-30

**Authors:** Hiroto Tanaka, Gen Li, Yusuke Uchida, Masashi Nakamura, Teruaki Ikeda, Hao Liu

**Affiliations:** 1 School of Engineering, Tokyo Institute of Technology, Tokyo, Japan; 2 Yokohama Institute for Earth Sciences, Japan Agency for Marine-Earth Science and Technology, Yokahama, Japan; 3 Graduate School of Engineering, Chiba University, Chiba, Japan; 4 Teral Inc., Fukuyama, Japan; Coastal Carolina University, UNITED STATES

## Abstract

Dolphins are well known as excellent swimmers for being capable of efficient cruising and sharp acceleration. While studies of the thrust production and power consumption of dolphin swimming have been the main subject for decades, time-varying acceleration process during successive fluke beats still remains poorly understood. In this study, we quantified the time-varying kinematics of a dolphin (*Lagenorhynchus obliquidens*) by directly recording its burst-accelerating swimming before vertical jump in an aquarium with two synchronized high-speed video cameras. We tracked the three-dimensional trajectories of its beak, body sides, and fluke. We found that dolphin could quickly accelerate from 5.0 m s^-1^ to 8.7 m s^-1^ merely by 5 strokes (i.e. 2.5 fluke beats) in 0.7 seconds. During the strokes, it was further found that the dolphin demonstrated a great acceleration in downstroke but less acceleration or even a slight deceleration in upstroke. Hydrodynamic forces and thrust power for each stroke were further estimated based on the equation of body motion and a static hydrodynamic model. The drag coefficient of the dolphin was estimated through computational fluid dynamics (CFD) modeling of the steady flows around a realistic geometric model based on 3-D scan data. The thrust and thrust power were then calculated by combining the body kinematics and the drag coefficient, resulting in a maximum stroke-averaged thrust and power-to-mass ratio of 1.3 × 10^3^ N and 90 W kg^-1^ at downstroke, and 3.3 × 10^2^ N and 19 W kg^-1^ at upstroke, respectively. Our results point out the importance of asymmetric kinematics in burst acceleration of dolphin, which may be a useful mechanism for biomimetic design of high-performance underwater robots.

## Introduction

Dolphins are well known for their excellent swimming ability, as demonstrated by high-speed swimming, porpoising, acrobatic jumping, and tail standing. In particular, dolphins demonstrate remarkably rapid acceleration from low speed to top speed. The hydrodynamics and energetics behind their high-speed swimming performance has attracted broad attention of both scientists and the public over decades. The famous “Gray’s paradox” was proposed in 1936, in which Sir James Gray calculated that the power output per kilogram of muscle of a dolphin during high-speed swimming was 7 times larger than that of a human [1]. Based on this result, Gray also suggested that the boundary layer around the dolphin may feature laminar instead of turbulent flow, thus reducing fluid drag. Gray’s paradox led to numerous follow-up studies in an attempt to elucidate the hydrodynamics of dolphin swimming [2–12]. To date, however, no evidence of the laminarization of a boundary layer or other special mechanism reducing fluid drag has been found, according to thorough reviews by Fish and Rohr (1999) [13] and Fish (2006) [14]. In fact, Gray’s calculation was flawed due to his underestimation of human power output. Gray calculated the thrust of the dolphin using the observed speed of 10.1 m s^-1^ for a 7 s sprint, whereas the power output of oarsmen during 3–5 min of sustained exercise was his reference for human performance. Since the power output of muscle decreases with time, the power output of a human for less than 10 s of exercise must be greater than Gray’s estimate based on 3–5 min of sustained exercise. Moreover, Gray used the power per unit kg mass of muscle as an index for comparison; however, the muscle mass and mechanics between muscle activity and locomotive movement are challenging to evaluate.

Although Gray’s paradox is evidently false, the hydrodynamic propulsion mechanism is still uncertain. One challenge associated with this argument is to acquire accurate kinematic and hydrodynamic data to determine a reliable estimate of the power output. There have been several previous studies reporting reliable maximum speeds measured using systematic methods. Lang and Daybell (1963) [7] measured a top speed of 7.7 m s^-1^ for *Lagenorhynchus obliquidens* swimming in a straight towing tank (96 m in length, 3.7 m in width, and 2.0 m in depth). In an open-ocean lagoon (300 m by 35 m in size, 3 m in depth), a maximum speed of 8.3 m s^-1^ for *Tursiops truncatus gilli* swimming in a 61-m course was reported by Lang and Norris (1966) [8]; 11.05 m s^-1^ for *Stenella attenuata* swimming in a 25-m course was reported by Lang and Pryor (1966) [15]. In the latter report, the drag-area coefficient (i.e., the drag per dynamic pressure) was also estimated from the deceleration during coasting after passing the goal line. The results, however, varied from 0.00288 to 0.00483 m^2^ due to inaccuracy in calculating the deceleration from distance and time data. Rohr et al. (2002) measured the swimming speed of different dolphin species in both an aquarium and the wild [16]. The maximum speed of *Tursiops truncatus*, *Delphinus delphis*, and *Paseudorca crassidens* swimming along the perimeter of a pool of dimensions 38 m by 15 m were 8.2, 8.0, and 8.0 m s^-1^, respectively. The maximum speed of *Tursiops truncatus* just before performing a vertical leap in the aquarium was measured to be 9.7 (SD (standard deviation) 0.8) m s^-1^. The maximum speed of free-ranging *Delphinus capensis* filmed from an airplane at altitude of 120–145 m ranged from 5.6 to 6.6 m s^-1^.

On the contrary to the speed measurement described above, time-varying acceleration for each stroke has been poorly investigated. Videler and Kamermans (1985) [17] recorded cruising *Tursiops truncatus* and *Sotalia guianensis* at less than 3.17 m s^-1^ in a straight tank with a single cine camera operating at 50 fps providing sequential lateral images during two fluke beats. Interestingly, the dolphins accelerated during downstrokes but decelerated during upstrokes in all cases. More recently, Fish et al. (2014) [18] conducted the first digital particle image velocimetry (DPIV) measurement quantifying the flow field around or behind the fluke of *Tursiops truncatus* when starting at less than 3.4 m s^-1^ during several fluke beats. Air bubbles were used as tracer particles in their study. It was reported that each fluke beat (i.e. downstroke and upstroke, or upstroke and downstroke) produced a pair of counter-rotating vortices. That implies fluid dynamic force was generated by both downstroke and upstroke, while the direction of the force was not identified. Acceleration during each stroke (i.e. downstroke or upstroke) in high-speed swimming, however, has never been measured so far. The technical challenge of the measurement was to capture the swimming motion with sufficient resolution for successive fluke beats resulting in long travel distance.

In this research, we aim to deepen the understanding on the propulsive mechanism in dolphins with more accurate measurements and estimations by utilizing advanced equipment and numerical approach. In particular, we focused on their acceleration performance per stroke. We measured a 3-D (three-dimensional) trajectory and time-varying acceleration of a dolphin, *Lagenorhynchus obliquidens*, in rapidly accelerating swimming during 7 successive fluke beats (i.e. 14 strokes). The dolphin accelerating for 2.2 s into a vertical high jump in an aquarium was recorded with two synchronized high-speed video cameras operating at 500 fps, enabling reconstruction of the 3-D trajectory of the dolphin from start to finish. We also estimated its thrust and thrust power based on equation of motion and a steady hydrodynamic model, where the drag of the dolphin was calculated from a computational fluid dynamics (CFD) simulation using a realistic 3-D rigid model. To obtain the morphological parameters and 3-D geometry, another *Lagenorhynchus obliquidens* was scanned with a portable non-contact 3-D scanner, and an accurate 3-D geometric model was created. The drag coefficients were then calculated in a conservative manner via CFD simulations using the static 3-D model. By combining the drag coefficient with the velocity and acceleration data, temporal thrust and thrust power were calculated from equation of motion. That made it possible to analyze the dolphin’s velocity, acceleration, and thrust generation in each stroke. The results demonstrated that the dolphin mainly accelerated and generated thrust in its downstrokes while less accelerated or slightly decelerated with less thrust in its upstrokes in the burst acceleration swimming even at high speed more than 5 m s^-1^ up to more than 8 m s^-1^. Although our estimation of thrust was assumed to be conservative due to the steady CFD simulations with the rigid model, the resultant maximum stroke-averaged thrust exceeded previously-reported time-averaged values for relatively long duration.

## Methods

### High-speed video recording of a swimming dolphin

**Fig 1:**
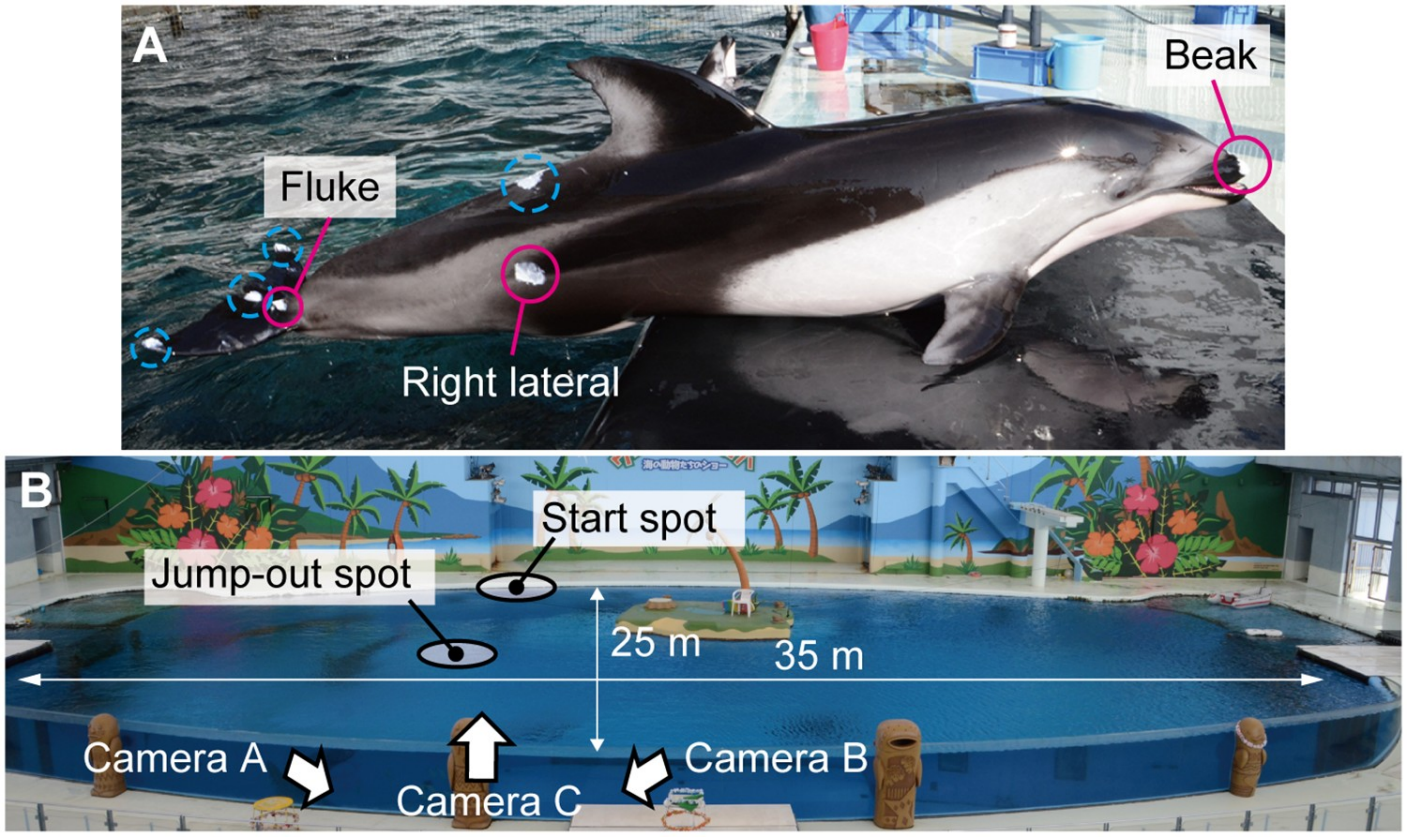
Video recording setup. **(A)** The Pacific white-sided dolphin (*Lagenorhynchus obliquidens*) recorded in this study. White ointments were painted as markers for motion tracking. Beak, lateral, and fluke markers indicated by solid magenta circles were used in this study, while the other markers indicated by dotted cyan circles were not used. **(B)** Overview of the water tank at Yokohama Hakkeijima Sea Paradise. The water depth is 6 m. The high-speed video cameras do not appear in this photo. Instead, positions and directions of the cameras are indicated with white arrows. The floating island in this photo was removed when the dolphin performed the jump.

We recorded the motion of a female Pacific white-sided dolphin (*Lagenorhynchus obliquidens*) performing a vertical high jump in an aquarium (Yokohama Hakkeijima Sea Paradise, Yokohama, Japan) on December 14, 2012. The dolphin was 2.1 m in length and had a mass of 110 kg. White ointment was painted on its body as markers for motion tracking (Fig 1(A)). The approximate size of the water tank was 35 m by 25 m, and the depth of the water was 6 m (Fig 1(B)). The water temperature was 23 °C. The animal welfare and housing condition details of the dolphin are provided in S1 Appendix.

The swimming of the dolphin proceeded as follows. The dolphin initially remained at rest, poking its beak from the water surface at the ‘start spot’ shown in Fig 1(B). Upon a signal from a human trainer standing in front of the dolphin, the dolphin leaped out of the water at that location and then dived again into the water. The dolphin descended toward the bottom of the tank and then ascended toward the water surface near the ‘jump-out spot’ shown in Fig 1(B), followed by an almost-vertical jump. A target ball was hung above the jump-out spot to encourage the dolphin to maximize its jump height. The dolphin hit the ball with its beak at the top of the jump. This swimming routine was performed as part of exhibition at the aquarium. Our recording was conducted during one of the training sessions between the exhibitions. The recorded dolphin always started from the same start spot and leaped from the same jump-out spot. The height of the target ball was determined by the aquarium for the exhibition to show the highest jump. The dolphin was daily trained to reach the ball and the height of the ball was raised to the limit through the trainings.

Note that we measured only a single swim by this specific individual dolphin due to practical reasons in the aquarium circumstances. Although it is possible that the swimming motions, which are presented in the Results section, vary with trials and individuals, owing to the daily repeating training and exhibition, the swimming routine of the dolphin was expected to be relatively stable. In fact, Rohr et al. (2002) measured the swimming speed of three *Tursiops truncatus* just before performing a total of 47 vertical jumps in an aquarium with a single underwater video camera of 60 fps, and the obtained maximum speed varied from 8.2 to 11.2 m s^-1^, with a mean of 9.7 (SD 0.8) m s^-1^ [16].

**Fig 2:**
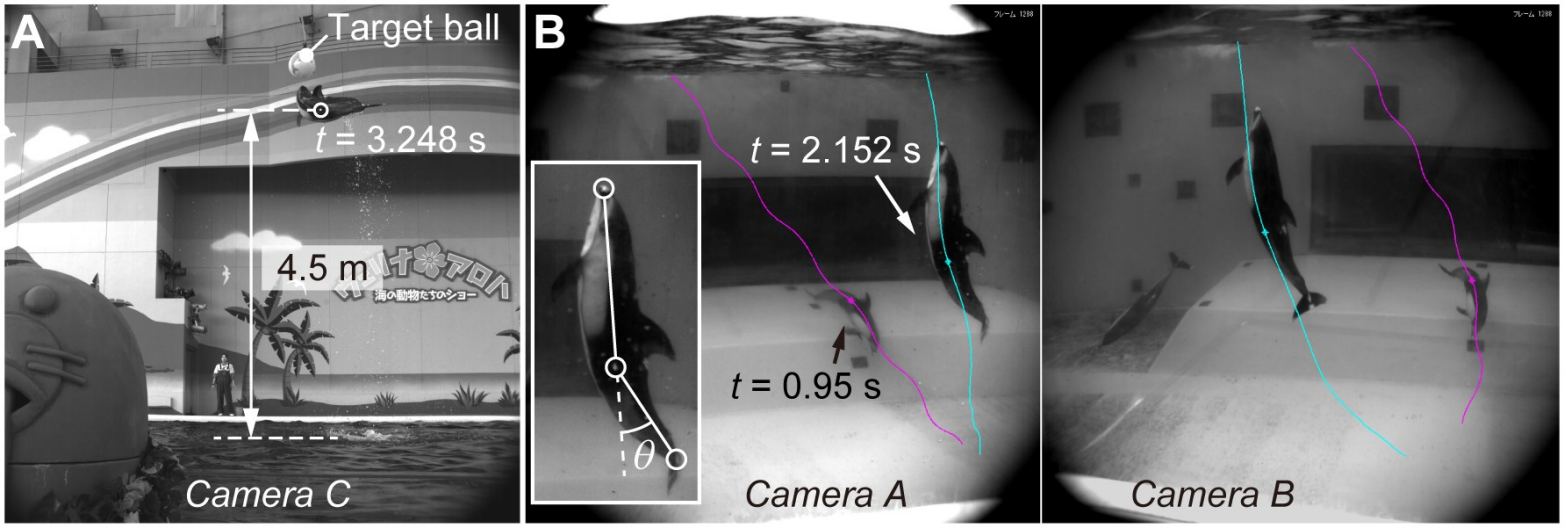
Example images captured by the high-speed cameras. **(A)** Captured image by Camera C showing the estimated height of the left lateral marker at *t* = 3.248 s. **(B)** Overlaid images captured by Camera A and B at *t* = 0.95 s and 2.152 s. The trajectories of the right and left lateral markers are illustrated in magenta and cyan, respectively. See also S1 and S2 Movies.

The underwater motion of the dolphin was recorded with two synchronized high-speed video cameras (SA3, Photron Ltd, Japan) through a transparent acrylic wall of the water tank, as indicated by Cameras A and B in Fig 1(B). A third high-speed video camera (SA2, Photron Ltd, Japan), which was also synchronized, was used to observe the dolphin in the air (Camera C in Fig 1(B)). One of the cameras were chosen as a master camera to which the other cameras were connected with trigger and sync cables, realizing synchronization for each frame. The frame rate of the cameras was set to 500 fps, and the resolution of the SA3 and SA2 were 1024 by 1024 pixels and 2048 by 2048 pixels, respectively; in addition, 25 mm CCTV lenses (B2512D, Pentax Corp., Japan) were used for all the cameras. Example images captured by these three cameras are shown in Fig 2. Size of the right lateral markers was around 13 cm corresponding to around 13 pixels in the beginning of the dive in the captured image. Size of the left lateral marker was around 5 cm corresponding to around 11 pixels before the jump out in the captured image. The maximum height of the left lateral marker was estimated to be approximately 4.5 m by taking the body length as a reference length scale (Fig 2(A)).

The experimental procedure was approved by Yokohama Hakkeijima Sea Paradise and Animal Experiment Committee of Chiba University.

### Camera calibration and 3-D motion analysis

3-D camera calibration was conducted taking into consideration refraction at the boundary between the water and the transparent wall using an algorithm proposed by Kwon [19], in which a geometric refraction-correction procedure and a 2-D DLT (direct linear translation) method were combined. The orientation and relative positions of the cameras and transparent walls of the water tank were measured using a laser distance meter (LS-511, MAX Co., Ltd., Japan). To obtain eight known reference points for the stereo camera calibration, a straight rope with two markers connecting the camera-sidewall and the opposite wall was photographed four times with changes to the connecting points.

The beak, right lateral, left lateral, and fluke markers were manually tracked in each image captured by the cameras using motion analysis software (Dipp-Motion 3D, Ditect Co. Ltd., Japan). The original images were in 12-bits greyscale. Manual tracking was implemented instead of the automated tracking, since the contrast of the images usually didn’t meet the threshold of the automated tracking software even with image adjustment of contrast and brightness. The 2-D trajectories of the lateral markers are shown in Fig 2(B). Note that the lateral and fluke markers were hidden by the body when turning near the bottom. Then, the 3-D coordinates of the lateral, beak, and fluke markers were calculated using each pair of images captured by the cameras. 3-D coordinate was not calculated when the marker was visible in only one of the cameras or not visible in the both cameras. The time series of the obtained 3-D coordinates were smoothed using a weighted 91-point weighted moving average with Hamming window designed to impose a 15 Hz cut-off frequency to eliminate noise due to the manual tracking.

Since the center of mass of the dolphin was unknown, the right or left lateral marker was used as a measured point for determining the position, velocity and acceleration of the dolphin in the following analysis. The velocity and acceleration were calculated using the second-order central difference method as follows:

$$v_{x}^{i}=\frac{x^{i+2}-x^{i-2}}{4\Delta t}$$ (1)

$$a_{x}^{i}=\frac{x^{i+2}-2x^{i}+x^{i-2}}{{\left(2\Delta t\right)}^{2}}$$ (2)

where *x*, *v*_*x*_, and *a*_*x*_ are the position, velocity, and acceleration in the *x*-direction, respectively. The upper suffix represents the discrete time index, and Δ*t* is the discrete time interval (0.002 s) derived from the frame rate of the high-speed cameras. By using i+2 and i-2 instead of i+1 and i-1 in Eqs (1) and (2), noise in tracking data could be reduced, resulting in smoother time-series data.

The downstroke and upstroke were distinguished by the body flection angle *θ*, which was defined as the angle between the beak-marker-to-lateral-marker vector and the lateral-marker-to-fluke-marker vector in three-dimensional space (Fig 2(B), bottom left). The timing of the start of each stroke was determined as the time when *θ* exhibited positive peaks. We distinguished between flexion to the ventral side and flexion to the dorsal side by visual observation. During the period when those markers were out of sight, the start times of the strokes were arbitrary decided via visual observation of the overall body flexion.

### Reconstruction of a realistic 3-D geometric model of a dolphin (*Lagenorhynchus obliquidens*)

**Fig 3:**
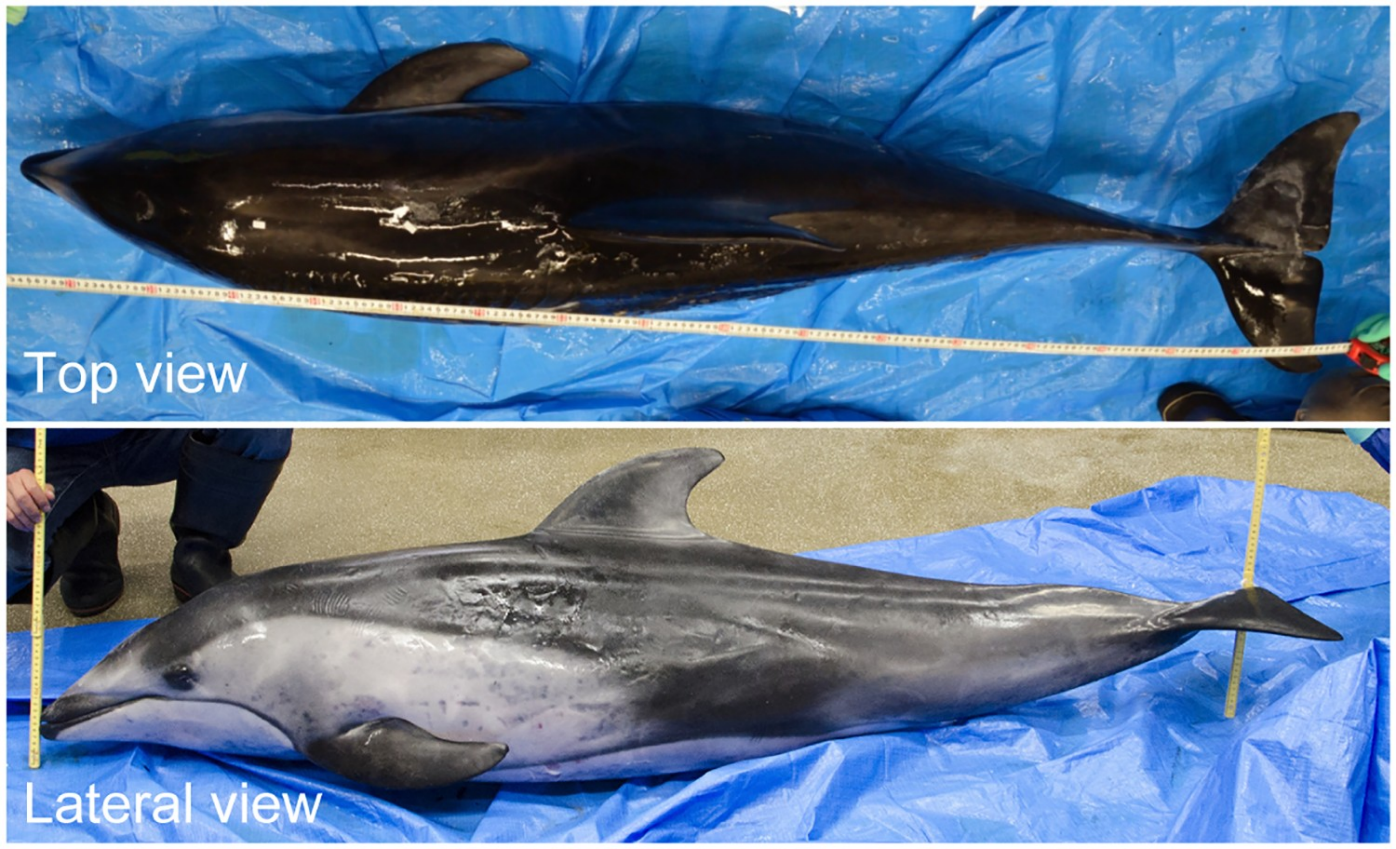
Frozen museum specimen of a Pacific white-sided dolphin (*Lagenorhynchus obliquidens*) scanned with a portable 3-D scanner.

**Fig 4:**
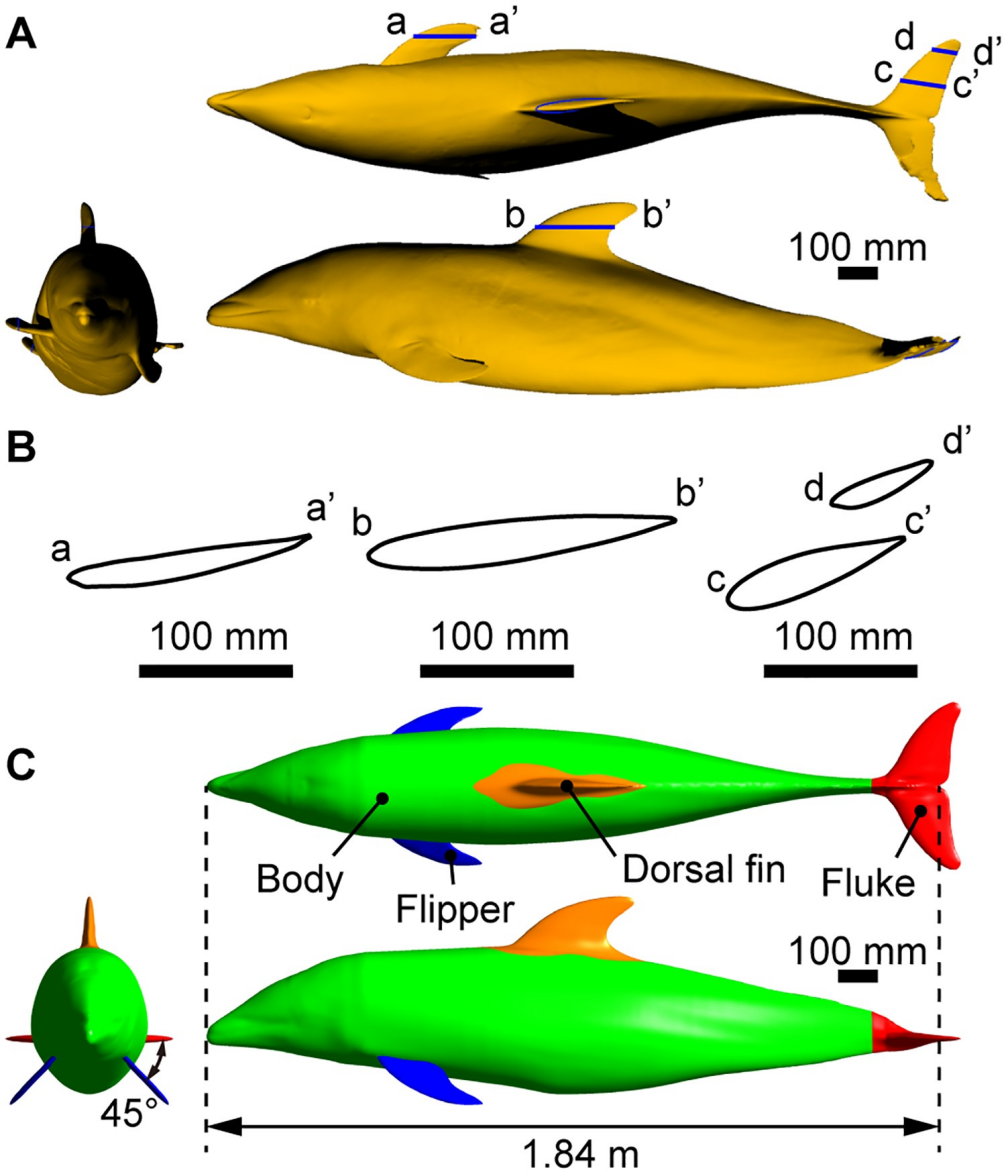
Dolphin models. **(A)** Original 3-D model of the frozen dolphin. **(B)** Representative cross-sectional shapes of the flippers, dorsal fin, and fluke of the original model. **(C)** Reconstructed symmetrical model used for the CFD simulation. The model was sectioned into four different parts: body, flippers, dorsal fin, and fluke. See also S1 and S2 Files for the models.

The fluid dynamic drag acting on the swimming dolphin was calculated using a CFD simulation. Since precise 3-D measurement on a living dolphin without any disservice was considered infeasible, instead, we measured a frozen museum specimen of the same species (*Lagenorhynchus obliquidens*) (Fig 3). This specimen had been found dead on the beach of Toyama Gulf in Japan and preserved in refrigerated storage by the National Museum of Nature and Science in Japan for inspection purposes. The frozen dolphin was 1.84 m in length and had a mass of 76 kg, which corresponded to 88% and 69% of the value for the measured live dolphin at the aquarium, respectively. We measured the 3-D surface shape with a portable non-contact 3-D scanner (Artec Eva, Artec 3D, Luxembourg) on April 14, 2014. The experimental procedure was approved by the National Museum of Nature and Science and Animal Experiment Committee of Chiba University. Created original 3-D model and its representative profiles of the flippers, dorsal fin, and fluke are shown in Fig 4(A) and 4(B). Since the shape of the body was distorted when frozen, we reconstructed a symmetric model of the dolphin for the CFD simulation from the original model using 3-D CAD software (Rhinoceros, Robert McNeel & Associates, USA) (Fig 3(B)).

The reconstruction process of the symmetric model was as follows:

1. The longitudinal axis was defined as the line connecting the beak and the middle of the trailing edge of the fluke.
2. The sagittal plane was defined such that the longitudinal axis lay on the sagittal plane and the dorsal fin was nearly aligned with the sagittal plane.
3. The original model was divided into four different parts: body, flippers, dorsal fin, and fluke.
4. Cross-sectional curves of the body perpendicular to the longitudinal axis were generated.
5. Each cross-sectional curve of the body was aligned such that the dorsal ridge of the cross section was aligned to the sagittal plane.
6. Each cross-sectional curve was divided by the sagittal plane into a left-side curve and a right-side curve. A mirror image of the curve on the right side was created on the left side. The median curve between the left curve and the mirrored curve was generated.
7. A mirror image of the median curve generated on the left side was generated on the right side. The mirrored median curve on the right side and the median curve of the left side were joined. The joint points were smoothed by adjusting the local curvature, and a symmetrical cross-sectional curve of the body was obtained.
8. A symmetrical body surface was generated as a smooth envelope over the symmetrical cross-sectional curves of the body.

The flippers, dorsal fin, and fluke were separately reconstructed into symmetrical profiles. The reconstruction procedure was similar to that of the body described above. For those appendages in pairs, the left flipper and the right half of the fluke in the original model were used for the reconstruction since their shapes were considered relatively natural. The reconstructed dorsal fin was aligned with the sagittal plane. The reconstructed fluke was perpendicular to the sagittal plane and parallel to the longitudinal axis. The reconstructed flippers were parallel to the longitudinal axis, and the angle between each flipper and body was set to 45 degrees (Fig 4(C)).

The morphological parameters of the models are summarized in Table 1, along with those of the live dolphin measured in the aquarium. The length of the dolphin was defined as the distance from the beak to the middle of the fluke. The reconstructed 3-D geometric models of dolphin are available in S1 and S2 Files to provide reference and resource for future researches.

**Table 1:** Morphological parameters of dolphins (*Lagenorhynchus obliquidens*) and 3-D models.

Sex	Condition, model	Length (m)	Mass (kg)	Volume (m^3^)	Surface area (m^2^)	Frontal projected area (m^2^)
Female	Frozen specimen, original model (Fig 4(A))	1.84 (87.6%)	76 (69.1%)	0.0763	1.44	0.107
-	Symmetrical model (Fig 4(B)	1.84 (87.6%)	74.4 (67.6%)*	0.0747	1.44	0.093
-	Scale-up symmetrical model	2.1 (100%)	111 (101%)**	0.111	1.87	0.121
Female	Live	2.1 (100%)	110 (100%)	-	-	-

*Calculated by scaling the mass of the frozen specimen with the volume ratio.

**Calculated by scaling the estimated mass of the symmetrical model with the volume ratio.

### CFD-based estimation of the dead drag coefficient

**Fig 5:**
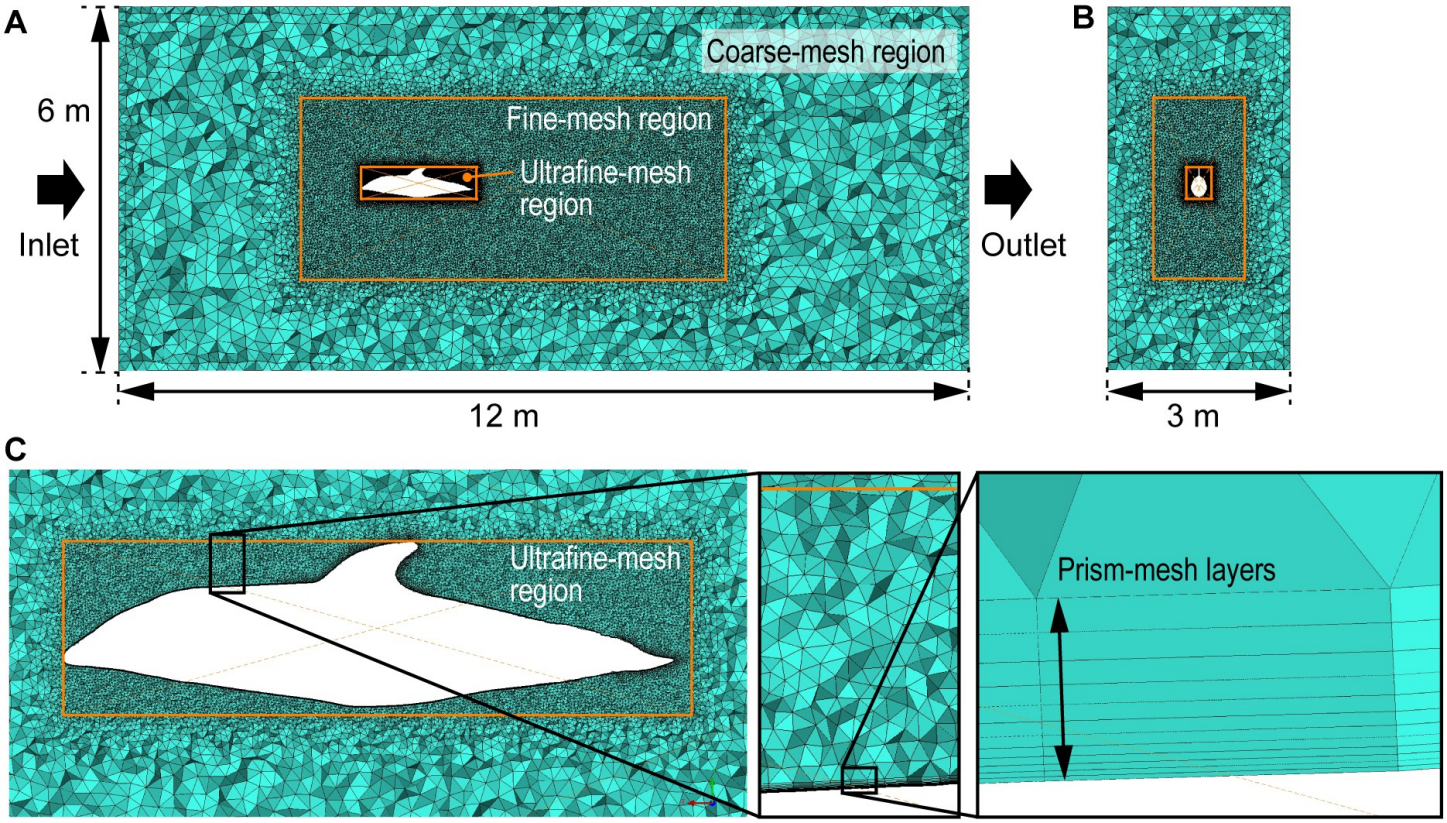
Meshes for CFD simulation. **(A)** Meshes at the sagittal plane of the whole fluid region. **(B)** Frontal cross section of the fluid region at the mid body. **(C)** Close-up of the meshes at the sagittal plane near the body surface.

A CFD simulation of the realistic 3-D model of dolphin in steady flow was performed using commercial finite volume method (FVM) CFD software (ANSYS CFX R14.5, ANSYS Inc., USA) to calculate the drag acting on the dolphin. The governing equations were the three-dimensional incompressible Reynolds-averaged Navier-Stokes equation and the continuity equation (i.e., conservation of mass), expressed by

$$u_{j}\frac{\partial u_{i}}{\partial x_{j}}=-\frac{1}{\rho }\frac{\partial p}{\partial x_{i}}+\frac{\mu }{\rho }\frac{\partial^{2}u_{i}}{\partial x_{j}\partial x_{j}}-\frac{\partial }{\partial x_{j}}\left(\bar{{u^{\prime }}_{i}{u^{\prime }}_{j}}\right)$$ (3)

$$\frac{\partial u_{j}}{\partial x_{j}}=0$$ (4)

where *i* and *j* are tensor notation suffixes, *u* and *x* are mean velocity and position vectors, *ρ* is density, *p* is mean pressure, and *μ* is viscosity. Riedeberger and Rist (2012) reported that the boundary layer of a dolphin model is turbulent even at cruising speeds (3 m s^-1^, *Re* = 1× 10^7^) based on CFD simulation with their *γ*-*Re*_*θ*_-transition turbulence model [20]. To address the expected turbulent boundary layer, we used a *k*-*ε* turbulence model with scalable wall function provided by the solver. The *k*-*ε* turbulence model is a type of Reynolds-averaged Navier-Stokes equation (RANS) turbulence model that is widely used for the gross estimation of flow fields [21]. The limitation of the RANS turbulence model is that unsteady vortices cannot be accurately simulated and may be underestimated [22]. The calculation volume was 12 m in length, 6 m in height and 3 m in width. Tetrahedral meshes and 12 layers of prism meshes adjacent to the dolphin model were automatically generated in the fluid region using meshing software, ICEM CFD, included with ANSYS (Fig 5). The fluid region was divided into 3 parts: coarse-mesh region, fine-mesh region, and the ultrafine-mesh region (Fig 5(A) and 5(B). The maximum size of mesh was set to 0.25 m for the coarse-mesh region, 0.05 m for the fine-mesh region, and 0.01 m for the ultrafine-mesh region. The thickness of the first prism layer adjacent to the dolphin model was set to 0.1 mm (Fig 5(C)). The layer thickness increased exponentially by a factor of 1.2, therefore the thickness of the outmost layer was 0.74 mm and the total thickness of the layers was 3.96 mm. The total number of the meshes was 7.7 × 10^6^ (Fig 5(B)). We used the same meshes for all *Re* cases. A constant longitudinal flow speed with 5% turbulence intensity was applied at the inlet boundary. At the outlet boundary, the average static relative pressure was set to 0 Pa. The other walls of the calculation volume were set as open boundaries. The angle of attack of the dolphin model, which was defined by the longitudinal axis of the body, was set to 0 degrees in all simulations.

The Reynolds number was defined as

$$Re=\frac{UL}{v}$$ (5)

where *U* (m s^-1^) is the applied constant flow speed, *L* (m) is a length of the dolphin, and *ν* (m^2^ s^-1^) is the kinetic viscosity of water at 23 °C (9.345 × 10^−7^ m^2^ s^-1^). We performed CFD simulations with *Re* of 1 × 10^6^, 1 × 10^7^, 2 × 10^7^, 3 × 10^7^, and 4 × 10^7^, which corresponded to 0.5, 5.1, 10.2, 15.2, and 20.3 m s^-1^, respectively.

The drag coefficient *C*_*D*_ was calculated as

$$C_{D}=\frac{D}{\frac{1}{2}\rho U^{2}S_{surface}}$$ (6)

where *D* is the calculated drag, *S*_*surface*_ is the surface area of the dolphin, and *ρ* (kg m^-3^) is the density of water at 23 °C (998 kg m^-3^). The friction drag coefficient, *C*_*D*, friction_, and pressure drag coefficient, *C*_*D*, pressure_, were similarly calculated based on the friction drag and pressure drag, which were separately output by ANSYS CFX. The drag originating from each body part (i.e., the body, flippers, dorsal fin, and fluke) was also evaluated. Note that the drag coefficient of the actively swimming dolphin is expected to be higher than that of the static model [14]. Oscillating body may reduce the thickness of boundary layer, leading to increase in friction drag up to factor of five [23]. Pressure drag may also increase due to deviation from the neutral streamlined shape when the dolphin bends its body. Moreover, our model was fully rigid unlike a real dolphin having a viscoelastic surface, which may also affect the resultant drag. Hence, our CFD model provided conservative estimation on the drag, while the actual drag on undulating dolphin should be higher.

In use of *k*-*ε* turbulence model with a wall function, *y*^+^ value at the first prism layer should be within the range of logarithmic law of velocity profile [24] which ranges from around 30 to 300 [25]. Here, *y*^+^ is a dimensionless height from a wall expressed as

$$y^{+}=\frac{\sqrt{\tau_{w}/p}}{v}y$$ (7)

where *τ*_w_ (Pa) is a wall shear stress and *y* is a height from the wall. Thus, *y*^+^ can be determined after calculation of the wall shear stress from the CFD simulation result. In our simulations, *y*^+^ was within the range of 30 to 110 in most locations on the surfaces when *Re* was 2 × 10^7^ or more, while *y*^+^ was below 30 in most locations when *Re* was 1 × 10^7^ and 1 × 10^6^ (S1 Fig). For the too small *y*^+^ less than 11, however, the scalable wall function we used automatically eliminated them from the logarithmic calculations.

We performed mesh-sensitivity investigation at *Re* of 2 × 10^7^ as follows. Firstly, the maximum size of the coarse-mesh of 0.25 m was compared with that of 0.125 m. The resultant drag for the 0.125-m case differed from that for the 0.25-m case only by 0.2%, that is, the 0.25-m coarse mesh was sufficiently fine. Secondly, the maximum size of the ultrafine-mesh of 0.01 m was compared with that of 0.02 m, since the 0.005-m case diverged. The resultant drag for the 0.01-m case was smaller than that for the 0.02-m case only by 1.2%. Hence it can be said that the 0.01-m ultrafine mesh was almost converged. Thirdly, the maximum size of fine mesh was decreased to 0.03 m from 0.05 m, resulting in decrease in drag only by 0.12%. Thus, the 0.05-m fine mesh was confirmed to be sufficiently fine. Finally, the number of prism layer was increased from 12 to 17, that reduced the drag only by 0.9%. Thus, we employed the 12 prism layers.

### Modeling of burst swimming and calculation of drag, thrust and power

The total instantaneous force acting on the swimming dolphin was calculated by multiplying the mass of the dolphin, *M*, and the calculated acceleration, as described in the Methods section. The thrust component in the direction of travel (i.e., direction of the velocity), *T*, was calculated by solving the equations of motion in the direction of the velocity:

$$M\vec{a}\cdot \frac{\vec{v}}{\left|\vec{v}\right|}=T+(M-\rho V)\vec{g}\cdot \frac{\vec{v}}{\left|\vec{v}\right|}-\frac{1}{2}\rho {\left|\vec{v}\right|}^{2}S_{surface}C_{D}(\left|\vec{v}\right|)-M_{AM}\vec{a}\cdot \frac{\vec{v}}{\left|\vec{v}\right|}$$ (8)

where *M* is the mass of the dolphin, $\vec{a}$ is an acceleration vector, $\vec{v}$ is a velocity vector, $\vec{g}$ is the acceleration due to gravity, *V* is the volume of the dolphin, $C_{D}(\left|\vec{v}\right|)$ is a drag coefficient dependent on $\left|\vec{v}\right|$, $\rho V\vec{g}$ is the buoyancy force, and *M*_AM_ is an added mass. Then, *T* was obtained by subtracting the buoyancy and gravity forces term ($(M-\rho V)\vec{g}\cdot \frac{\vec{v}}{\left|\vec{v}\right|}$) from the sum of the total force term ($M\vec{a}\cdot \frac{\vec{v}}{\left|\vec{v}\right|}$), drag term ($\frac{1}{2}\rho {\left|\vec{v}\right|}^{2}S_{surface}C_{D}(\left|\vec{v}\right|)$), and added-mass drag term ($M_{AM}\vec{a}$).

*M* was directly measured by weighing the dolphin on shore in the aquarium. *S*_surface_ and *V* of the dolphin in the aquarium were calculated by scaling up the measurements of the symmetric CFD model. Since the dolphin in the aquarium was 1.14 times larger than the symmetrical model in length, the scale factor was 1.14^2^ = 1.30 for the area and 1.14^3^ = 1.48 for the volume. In fact, scale-up mass was 74.4 kg × 1.48 = 110.1 kg which is almost equal to the measured mass of the live dolphin in the aquarium (110 kg) (Table 1), that supports the validity of morphological scaling. Thus, the surface area and volume of the dolphin in the aquarium were calculated to be 1.87 m^2^ and 0.111 m^3^, respectively (Table 1).

$C_{D}(\left|\vec{v}\right|)$ was calculated via linear interpolation of the calculated values output by the CFD simulation. Since the CFD simulation was steady with a rigid model, the calculated drag can be regarded as a virtual parasite drag to which the dolphin would be subject if the dolphin stopped beating and shifted to gliding with a static position. Note that the *C*_*D*_ could be underestimated as explained in the Method section, that leads to underestimate in calculation of *T*.

The added mass represents a virtual mass due to water surrounding the dolphin. As reviewed by Brennen (1982), the added mass of a prolate spheroid in non-viscous flow can be analytically expressed as follows [26]:

$$\begin{array}{llll} M_{AM}=\kappa \frac{4}{3}\rho \pi r_{a}^{2}r_{b}^{2}, & \kappa =\frac{\alpha }{2-\alpha }, & \alpha =\frac{1-e^{2}}{e^{3}}\left(\text{ln}\frac{1+e}{1-e}-2e\right), & e=\sqrt{\frac{r_{a}^{2}-r_{b}^{2}}{r_{a}^{2}}} \end{array}$$ (9)

where *r*_*a*_ is the major radius and *r*_*b*_ is the minor radius. To estimate the effect of the added mass in our study, we assumed a prolate spheroid with the same length and volume as our dolphin model (i.e. 2.1 m and 0.111 m^3^). Then, the obtained *r*_*a*_, *r*_*b*_, *e*, *κ*, and *M*_AM_ were 1.05 m, 0.16 m, 0.99, 0.039, and 4.3 kg, respectively. This value of added mass *M*_AM_ corresponds to 3.9% of *M*, that is, the added mass effect is not significant in our calculations.

Finally, the instantaneous power output by the thrust in the direction of travel, *P*, was calculated as

$$P=T\left|\vec{v}\right|.$$ (10)

Note that the calculation of *P* does not consider the work performed in the direction perpendicular to the velocity vector; thus, the actual power output may be greater than *P*.

The calculation of *T* and *P* was performed at each discrete time step using the values from Eqs (1) and (2). The stroke-averaged values and fluke-beat-averaged values were then calculated using the time-series values for *T* and *P*.

## Results

### Calculation of velocities and accelerations

**Fig 6:**
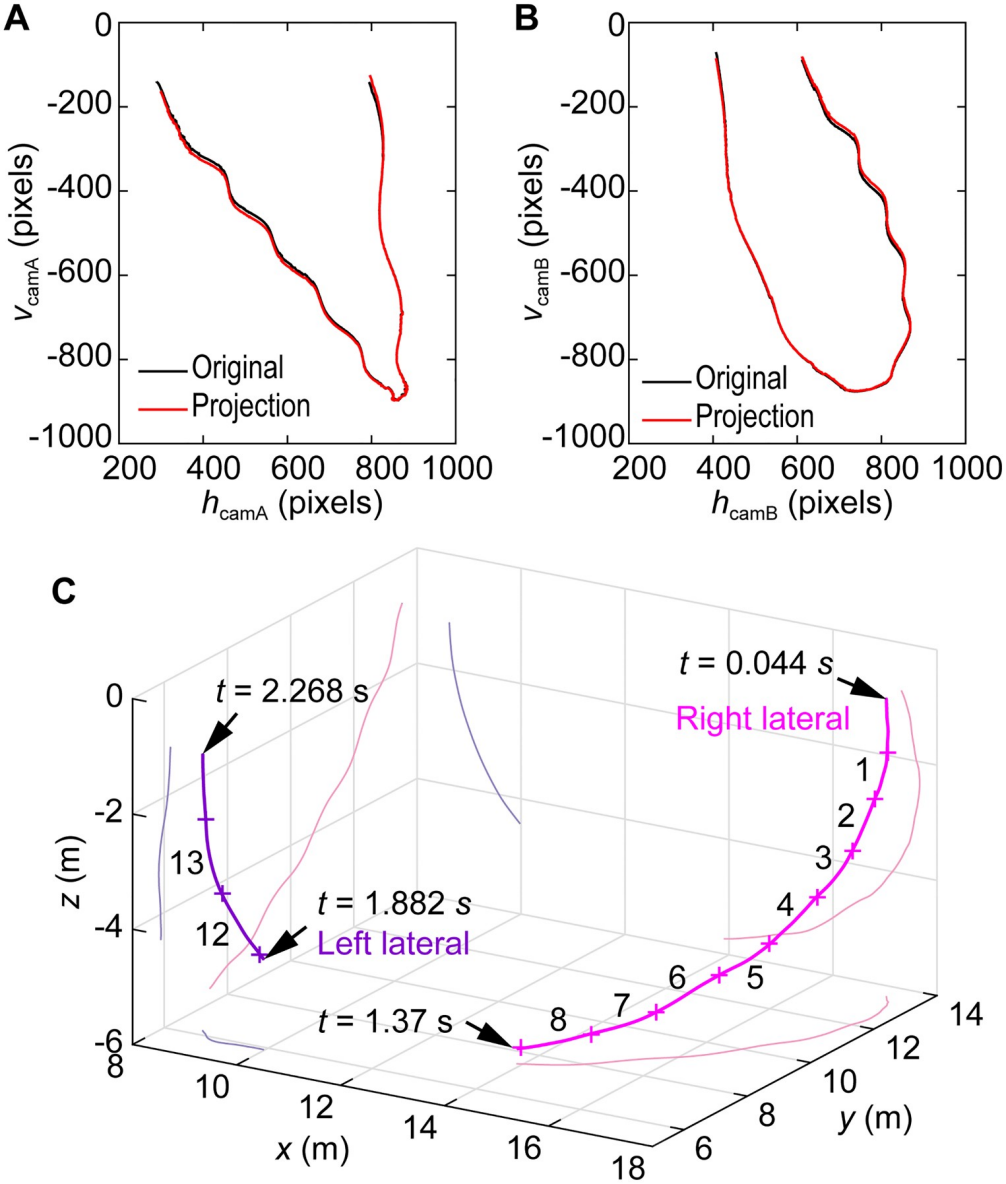
Trajectories of the markers. **(A, B)** Projection and original trajectories of the beak marker for Camera A and B. **(C)** 3-D trajectory of the right and left lateral makers of the dolphin with the stroke numbers. *t* = 0 s was the time when the entire body slipped underwater. The beak reached the water surface at *t* = 2.232 s. See also S1 and S2 Datasets for the 3-D coordinates before and after lowpass filtering.

To confirm the quality of the camera calibration, original trajectory and projection trajectory of the beak marker on image plane of each camera were compared. The projection trajectories were generated by projecting the calculated 3-D trajectories to the camera image planes. As shown in Fig 6(A) and 6(B), the projection trajectories well matched the original trajectories, indicating the validity of the camera calibration. Mean errors between the original and projected pixel coordinates on the image planes were 6.3 (SD 4.1) pixels for Camera A and 6.4 (SD 4.0) pixels for Camera B, respectively.

The obtained 3-D trajectories of the lateral markers are shown in Fig 6(C). The *z*-axis is vertical, and the origin is set at the water surface. The dolphin initially descended and turned right toward the direction of travel; it then ascended into its vertical jump. As shown in the supplemental videos, the dolphin rolled slightly in the counter-clockwise direction around its longitudinal axis, i.e., the left flipper faced the bottom surface of the tank, and the dolphin turned to its right-ventral side.

**Fig 7:**
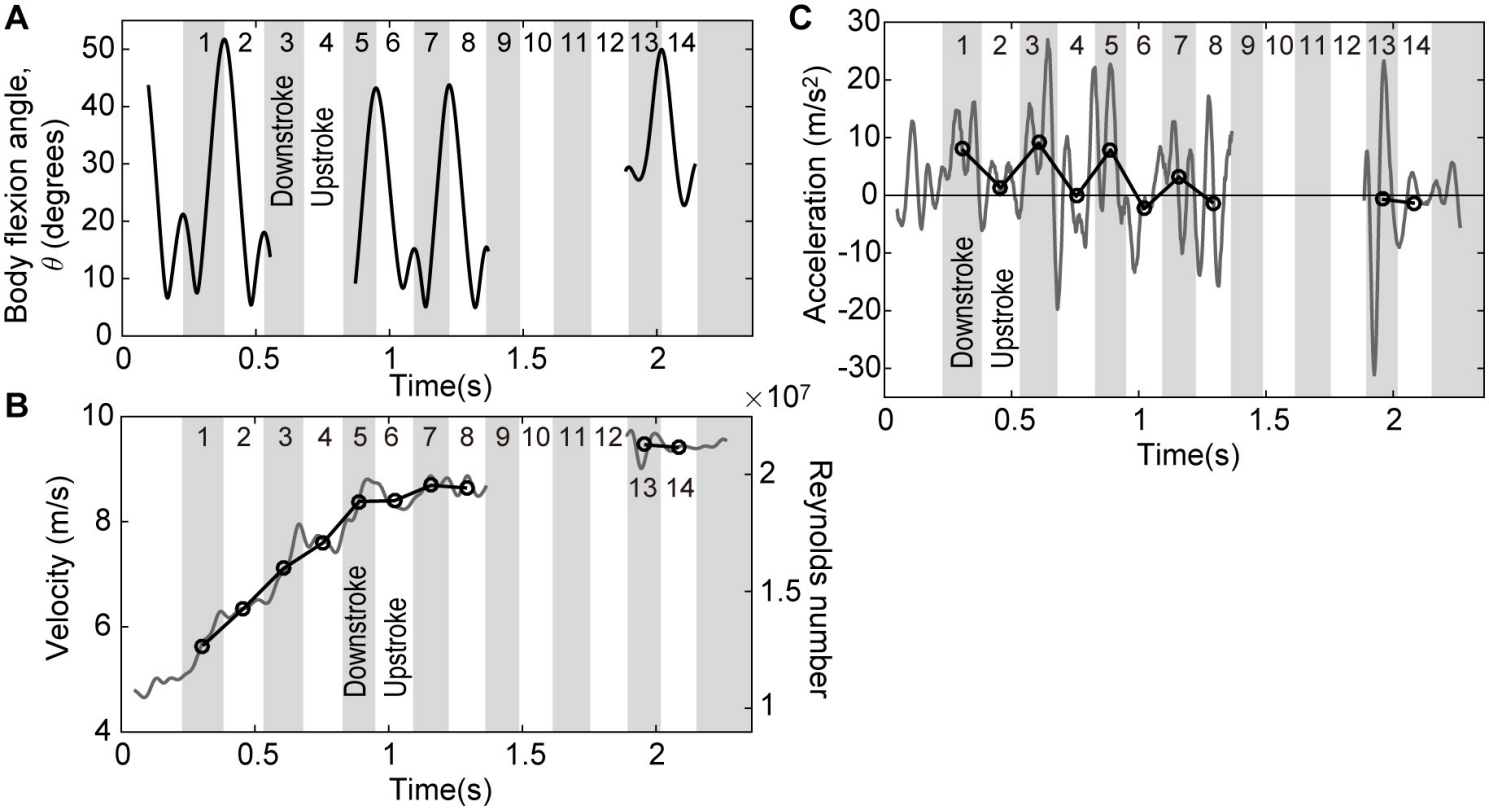
Time histories of the measured motion parameters. **(A)** The bending angle, *θ*. **(B)** Velocity and Reynolds number. **(C)** Acceleration. Open circles indicate stroke-averaged values. Strokes are numbered from 1 to 14. The fluke marker was out of sight during the 3rd, 4th, and 9-12th strokes. Velocity and acceleration data between the 9th stroke and the 12th strokes were excluded from the following analysis since the lateral markers were out of sight. See also S3 and S4 Datasets for the timings of key events and the time-series data of the velocity and acceleration, respectively.

The obtained bending angle, velocity, Reynolds number, and acceleration are shown in Fig 7. Strokes are numbered from 1 to 14, and the stroke-averaged values are also represented by open circles. *t* = 0 s corresponds to the moment when the dolphin’s entire body passing completely underwater, and *t* = 2.358 s corresponds to the time at which the left lateral marker reached the water surface. The first 45 data points and the last 45 data points were cropped due to the 91-point moving average used to smooth the position data, as explained in the Methods section. During the transition from descent to ascent, both the right and left lateral markers were hidden by the dolphin’s body since it swam toward the cameras. Thus, we excluded this period, ranging from the 9th to 12th strokes, from the following analysis and discussion.

The average fluke-beat cycle from the 1st to 14th strokes was 0.28 (SD 0.02) s, and the average fluke-beat frequency was 3.7 (SD 0.3) Hz. The average ratio of the downstroke duration to the fluke-beat cycle was 0.49 (SD 0.02), meaning that the durations of downstrokes and upstrokes were almost identical. The maximum ventral-side flexion was 52° at the end of the No.1 stroke (downstroke), and the maximum dorsal-side flexion was 30° at the end of the No.14 stroke (upstroke) (Fig 7(A)).

The stroke-averaged velocity rapidly increased during the first three fluke beats from 5.0 m s^-1^ at the start of the 1st stroke (*t* = 0.228 s) to 8.7 m s^-1^ at the end of the 5th stroke (*t* = 0.950 s) (Fig 7(B)). The maximum stroke-averaged velocity was 9.5 m s^-1^ at the 13th stroke (*t* = 1.949 s). The corresponding stroke-averaged Reynolds number ranged from 1.3 × 10^7^ to 2.1 × 10^7^. The terminal velocity can also be calculated from the dolphin’s body length and the duration when the body passed the water surface: the beak reached the water surface at *t* = 2.232 s, the trailing edge of the fluke reached the water at *t* = 2.466 s, the body length was measured on shore as 2.1 m, and then the velocity was calculated to be 9.0 m s^-1^. This value is 95% of the 14th stroke-average velocity of 9.4 m s^-1^. This difference is possibly due to deviation between the velocity vector of the left lateral marker and body longitudinal axis, deceleration by losing upward buoyancy force for the body part above the water surface, or measurement error of the body length on shore. Moreover, error in the 3-D camera calibration could be caused by measurement error of the camera positions, camera angles, or positioning error of the calibration markers due to slack in the ropes to which the calibration markers were attached.

It was found that the dolphin mainly accelerated forward with downstrokes rather than with upstrokes according to the stroke-averaged acceleration values (Fig 7(C)). The mean value of the stroke-average acceleration during downstrokes in the first three fluke beats (no. 1, 3, and 5) and the standard deviation was 8.3 (SD 0.7) m s^-2^ (0.85 (SD 0.07) G), whereas those during upstroke in the first three fluke beats were -0.4 (SD 1.8) m s^-2^ (0.04 (SD 0.19) G). In the last fluke beat (no. 13 and 14), the acceleration was almost zero during both the downstroke and upstroke.

### CFD-based flows and drag coefficients

**Fig 8:**
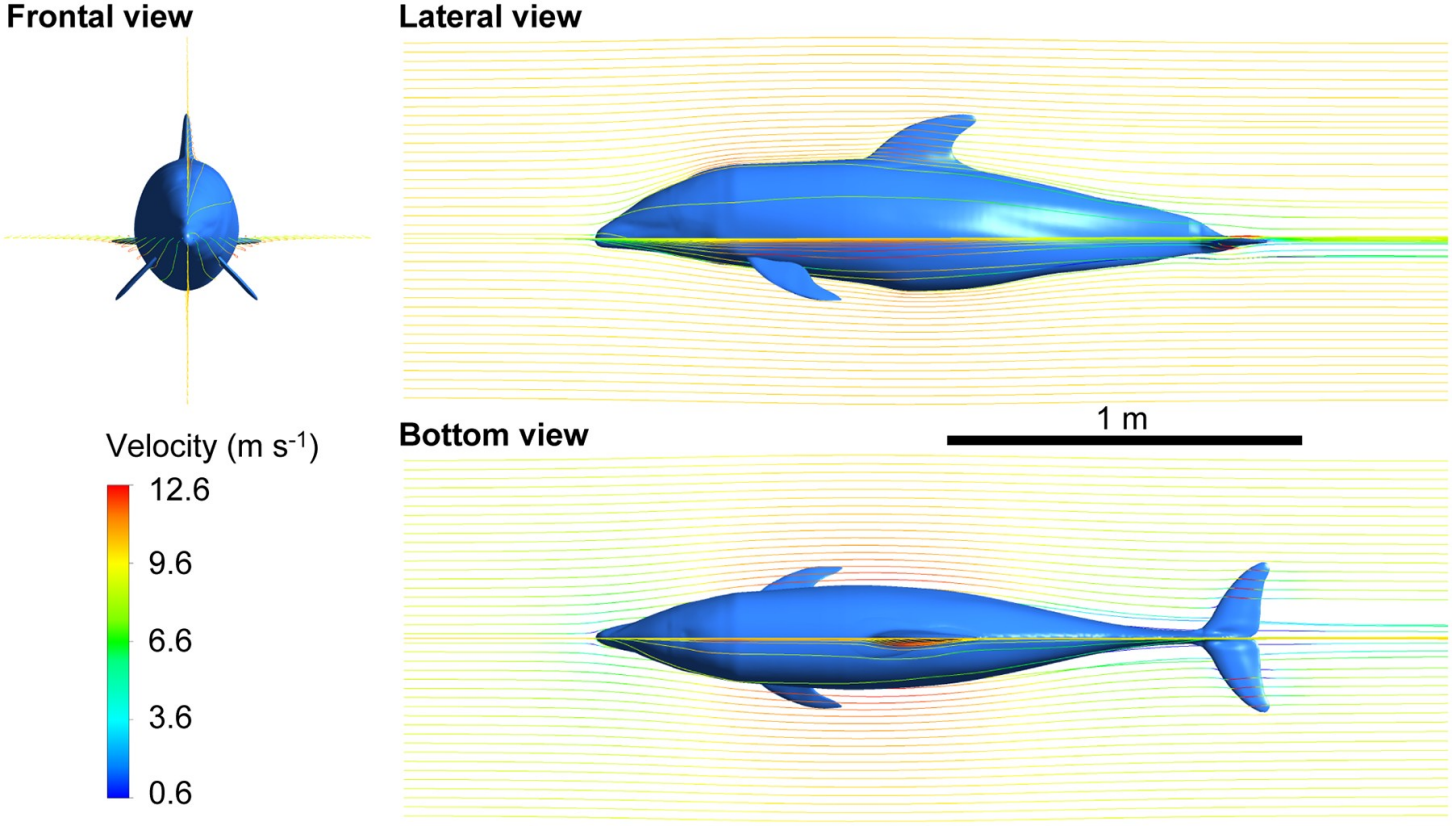
Streamlines starting from vertical and horizontal lines in the CFD calculation with the model of white-beaked dolphin (*Re* = 2 × 10^7^, *U* = 10.2 m s^-1^). The color of the streamlines indicates the velocity magnitude.

**Fig 9:**
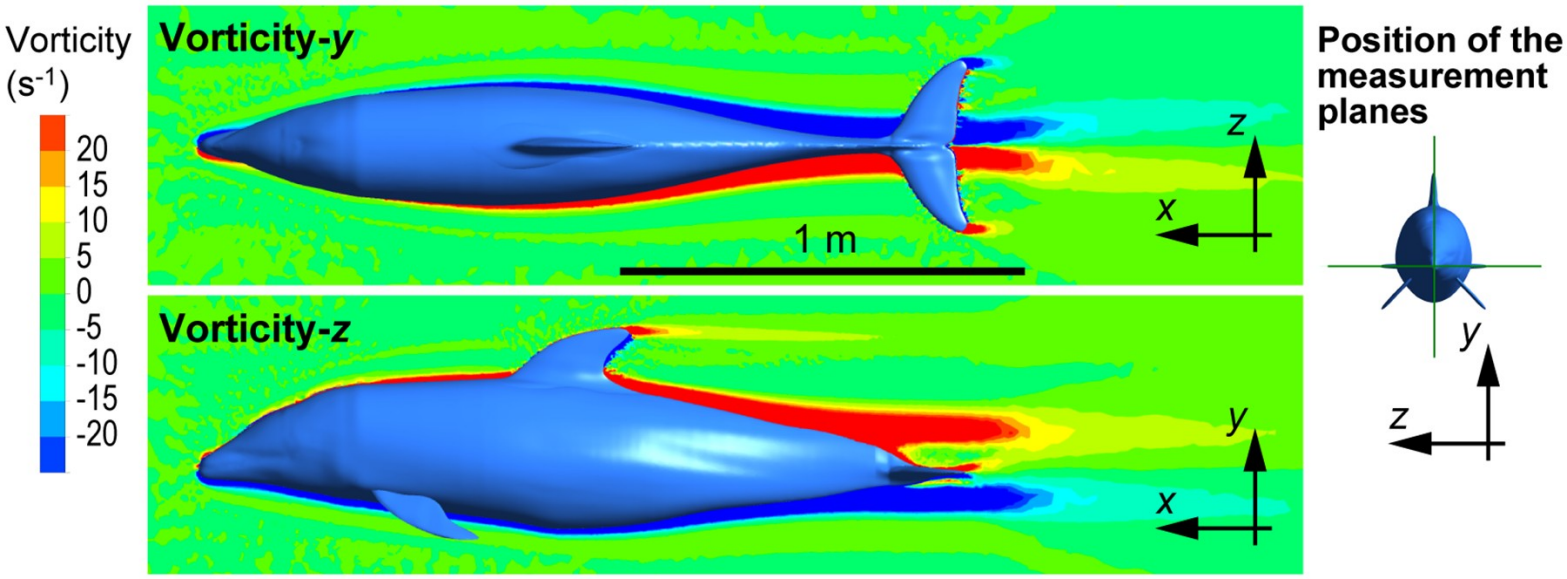
Vorticity about the *y*-axis on the horizontal plane (upper) and vorticity about the *z*-axis on the vertical plane (lower) around the model of white-beaked dolphin (*Re* = 2 × 10^7^, *U* = 10.2 m s^-1^).

Visualization of the streamlines around the 3-D model of the dolphin at *Re* = 2 × 10^7^ (i.e., *U* = 10.2 m s^-1^) demonstrates that the flow smoothly followed the dolphin’s surface with this posture during high-speed gliding (Fig 8). The flow speed decreased around the head and lower part of the body and increased around the middle of the body. The vorticity about the vertical axis and lateral axis for the same *Re* shown in Fig 9 illustrates that the thickness of the boundary layer started to increase in the middle of the body where body cross-section area peaks. Note that the steady-flow CFD calculation with the *k*-*ε* RANS turbulence model provides a gross time-averaged estimate, but it cannot accurately simulate unsteady vortices in the boundary layer and flow separation, as mentioned in the Methods section.

**Fig 10:**
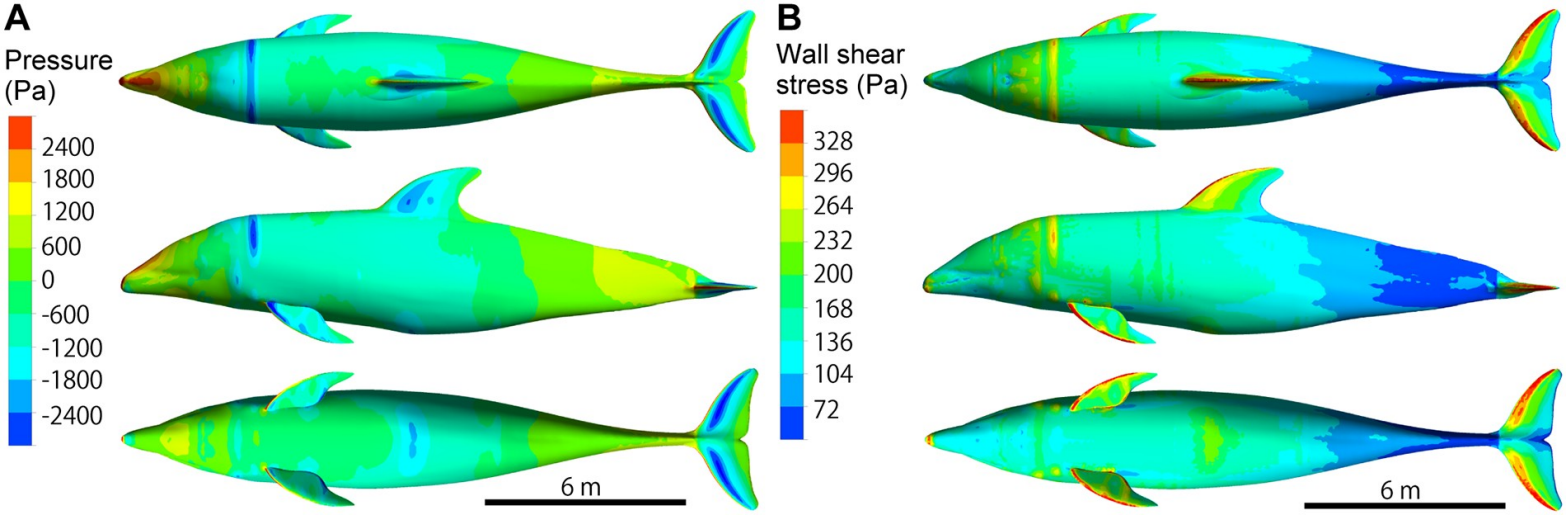
Color contour map visualizing the calculated hydrodynamic forces on the model of white-beaked dolphin (*Re* = 2 × 10^7^, *U* = 10.2 m s^-1^). (**A**) Pressure. (**B**) Wall shear stress.

The calculated pressure on the model surface demonstrated that the pressure was positive at the head and lower part of the body as a result of the decreased flow speed (Fig 10(A)), whereas the pressure on the middle part of the body was negative due to the increased flow speed. Note that 0 Pa was defined as the average static pressure at the outlet boundary. In contrast, the wall shear stress was large at the upper part of the body and decreased remarkably at the lower part of the body (Fig 10(B)). The wall shear stress was also high at each leading edge of the fins and flippers.

The calculated drag coefficients of the dolphin model for various *Re* are summarized in Table 2, along with the contributing ratio for each part. Assuming a rigid body in steady flow, it was found that both friction drag coefficient (*C*_*D*, friction_) and pressure drag coefficient (*C*_*D*, pressure_) considerably contribute to the total drag coefficient (*C*_*D*_). The contributing ratio of the friction drag to the total drag ranged from 72% to 63%, while that of the pressure drag ranged from 28% to 37%. It was also found that not only the body but also the other appendages (i.e., flippers, dorsal fin, and fluke) contributed to the drag production. The contributing ratio of the body ranged from 62% to 54%, while that of the other parts ranged from 38% to 46%.

**Table 2:** Calculated drag coefficients of the 3-D realistic model of dolphin and contributions of each part.

Re	*C*_*D*_	Friction *C*_*D*_	Pressure *C*_*D*_	*C*_*D*_ by body	*C*_*D*_ by flippers	*C*_*D*_ by dorsal fin	*C*_*D*_ by fluke
1 × 10^6^	6.1 × 10^−3^ (100%)	4.4 × 10^−3^ (72%)	1.7 × 10^−3^ (28%)	(61.7%)	(14.0%)	(9.5%)	(14.8%)
1 × 10^7^	4.7 × 10^−3^ (100%)	3.2 × 10^−3^ (67%)	1.6 × 10^−3^ (33%)	(57.1%)	(16.0%)	(10.2%)	(16.7%)
2 × 10^7^	4.3 × 10^−3^ (100%)	2.8 × 10^−3^ (65%)	1.5 × 10^−3^ (35%)	(55.5%)	(16.8%)	(10.4%)	(17.2%)
3 × 10^7^	4.2 × 10^−3^ (100%)	2.7 × 10^−3^ (64%)	1.5 × 10^−3^ (36%)	(54.6%)	(17.2%)	(10.6%)	(17.6%)
4 × 10^7^	4.0 × 10^−3^ (100%)	2.6 × 10^−3^ (63%)	1.5 × 10^−3^ (37%)	(54.0%)	(17.5%)	(10.7%)	(17.8%)

### Thrust forces and power output

**Fig 11:**
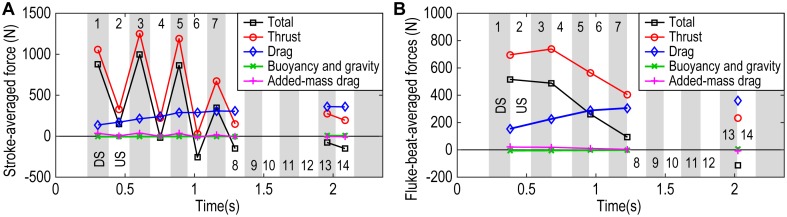
Calculated forces. **(A)** Stroke-averaged values. **(B)** Fluke-beat-averaged values. Grey region and white region represent downstroke and upstroke, respectively. See also S5 and S6 Datasets for time-series force and drag coefficient data and averaged force data, respectively.

The stroke-averaged thrust in the direction of travel calculated with Eq (6) is shown in Fig 11(A), along with other forces, such as the total force, drag and the sum of the gravitational and buoyancy forces. The calculated buoyancy force was 1075 N, which almost cancelled out the gravitational force of 1079 N, suggesting that the dolphin was close to being neutrally buoyant.

A remarkable asymmetry in thrust generation between downstrokes and upstrokes was found in the first half of the swimming until the 8th stroke: each downstroke generated greater thrust (from 6.7 × 10^2^ to 12.5 × 10^2^ N), than each upstroke (from 0.3 × 10^2^ to 3.3 × 10^2^ N). The maximum stroke-averaged thrust by downstroke corresponded to 116% of the dolphin’s weight (1079 N). In the last two (13th and 14th) strokes at top speed, the thrust values (2.8 × 10^2^ and 1.9 × 10^2^ N) were less than the drag values (3.6 × 10^2^ and 3.6 × 10^2^ N), resulting in negative total forces.

The fluke-beat-averaged thrust exhibited large positive values in the first two beats (Fig 11(B)). The maximum was 7.4 × 10^2^ N, which is 69% of the dolphin’s weight. The fluke-beat-averaged drag became comparable to thrust with increased velocity, increasing from 1.5 × 10^2^ to 3.6 × 10^2^ N.

**Fig 12:**
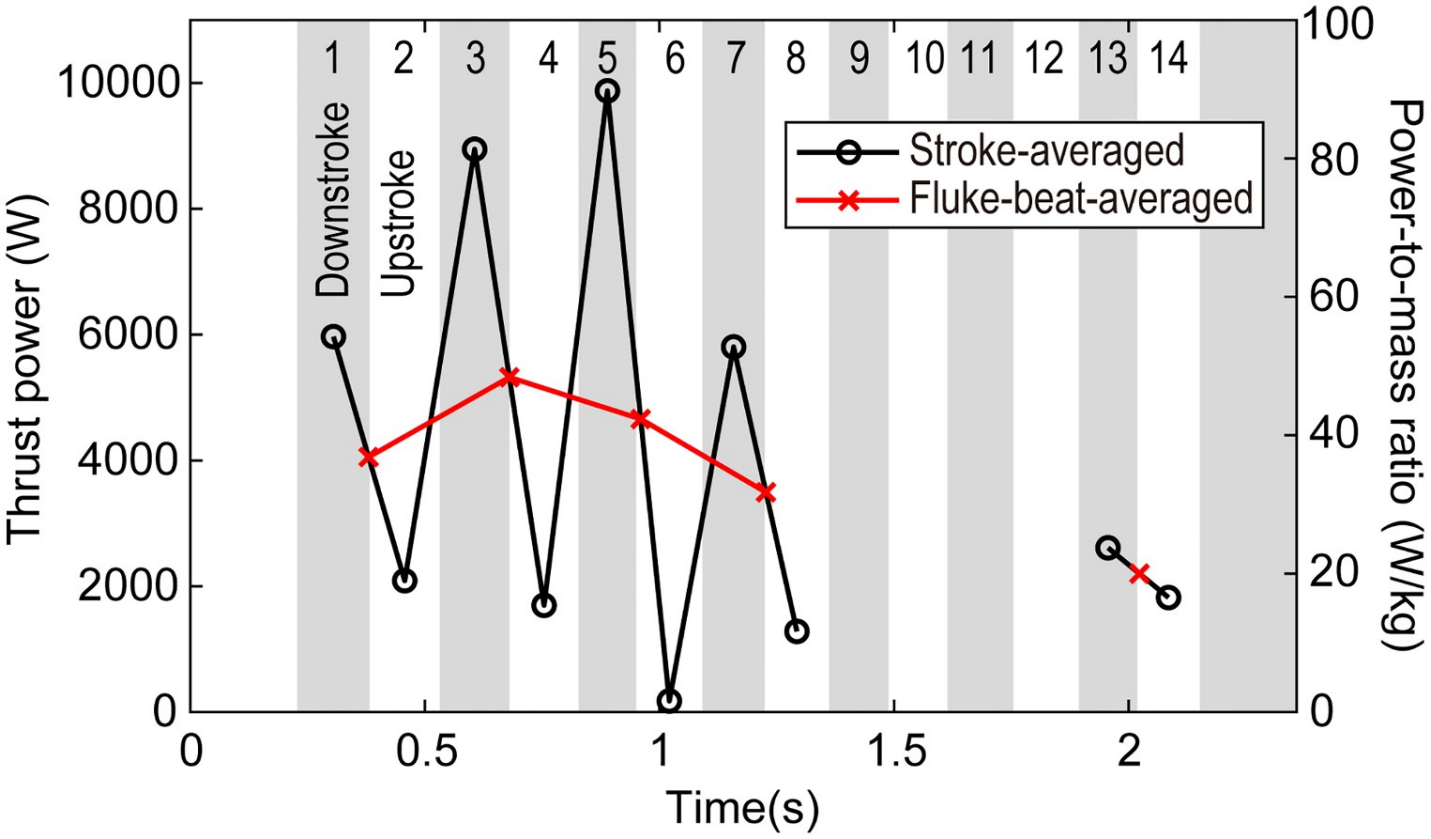
Stroke-averaged and fluke-beat-averaged thrust power and power-to-mass ratio. Grey region and white region represent downstroke and upstroke, respectively. See also S6 Dataset for each value.

The thrust power and power-to-mass ratio are shown in Fig 12. The stroke-averaged thrust power and the thrust during downstrokes were always greater than those during upstrokes. The thrust power during downstrokes ranged from 2.6 × 10^3^ to 9.9 × 10^3^ W, and those during upstrokes ranged from 0.2 × 10^3^ to 2.1 × 10^3^ W. Based on the mass of the dolphin (110 kg), the power-to-mass ratio was calculated to range from 24 to 90 W kg^-1^ during downstrokes and from 2 to 19 W kg^-1^ during upstrokes. Consequently, the fluke-beat-averaged thrust power and the fluke-beat-averaged power-to-mass ratio ranged from 2.2 × 10^3^ to 5.3 × 10^3^ W and from 20 to 48 W kg^-1^, respectively.

## Discussion

### Maximum swimming speed of *Lagenorhynchus obliquidens*

Fish and Rohr (1999) broadly summarized the swimming speeds of various dolphins, including both reliable and non-reliable data; two records were listed for *Lagenorhynchus obliquidens* for short-duration high-speed swimming [13]. One is by Lang and Daybell (1963) [7], who conducted speed tests of a 91-kg 2.0-m dolphin in a 97 m tank; the top speed was 7.7 m s^-1^ which was 3.85 BL s^-1^ where BL is its body length. Another is by Ridgway and Johnston (1966) [27], who reported that the dolphin could overtake their boat at speeds no faster than 7.2 m s^-1^. The maximum stroke-averaged speed in the present study was 9.5 m s^-1^ (4.5 BL s^-1^) at the 13th stroke (*t* = 1.949 s), that exceeds those previous records.

### Drag coefficients

**Fig 13:**
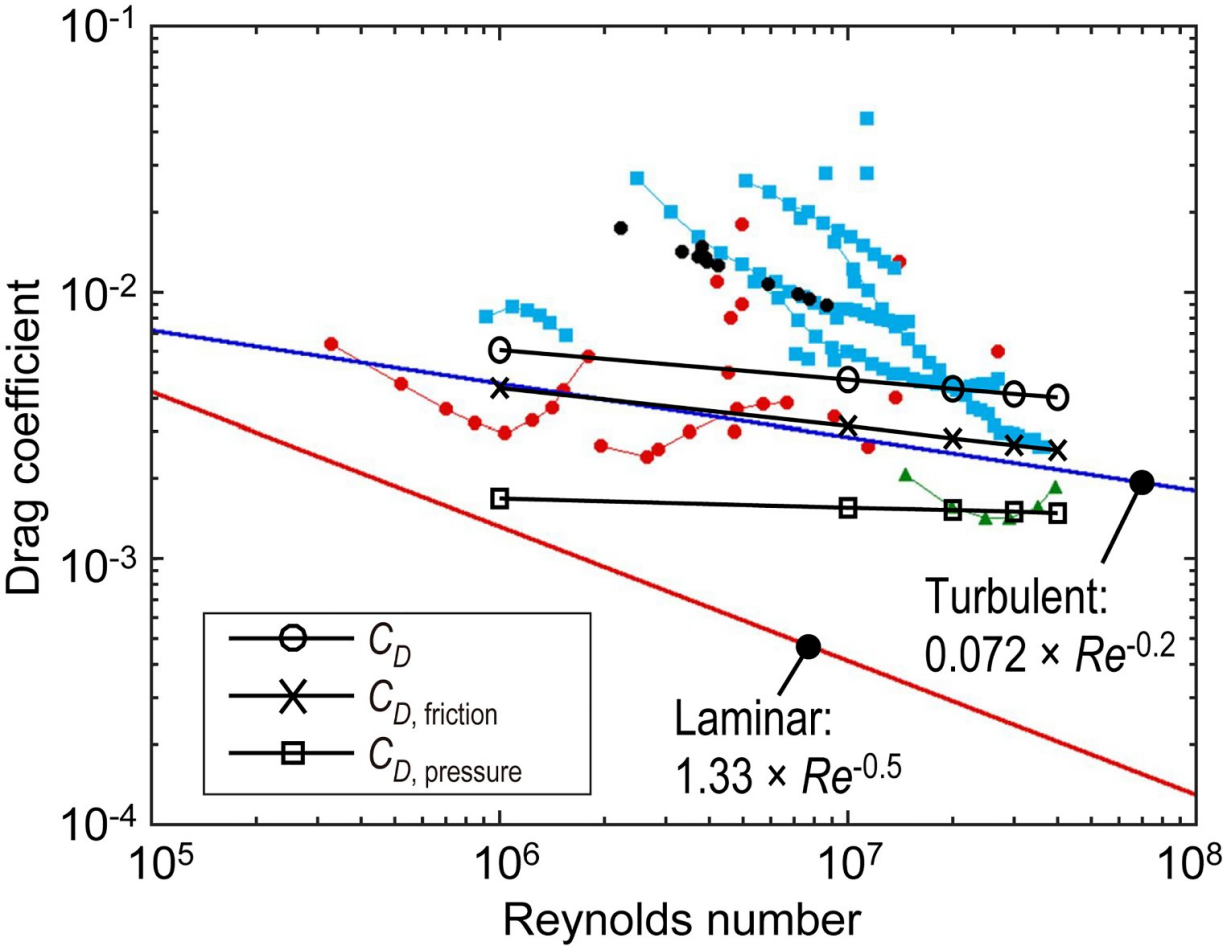
Calculated drag coefficients, *C*_*D*_, (open circles), contribution of friction drag to *C*_*D*_ (open diamonds), and contribution of pressure drag to *C*_*D*_ (open square) against Reynolds number superimposed on the previous data summarized by Fish (2014) [18]. The blue line represents the frictional drag coefficient for a flat plate with turbulent boundary layer flow, and the red line represents a flat plate with laminar boundary flow. The data were obtained from experiments on rigid models, towed bodies and gliding animals (red solid circles), from hydrodynamic models based on swimming kinematics (blue squares), from a rigid ‘dolphin’ model with the shape of a solid of revolution of the NACA 66 series (green triangles), and from the calculation based on the results of DPIV measurements (black solid circles). The data are from Lang and Daybell (1963) [7]; Lang and Pryor (1966) [15]; Aleyev and Kurbatov (1974) [3]; Kayan (1974) [6]; Purves et al. (1975) [10]; Webb (1975) [11]; Aleyev (1977) [2]; Chopra and Kambe (1977) [4]; Yates (1983) [12]; Videler and Kamermans (1985) [17]; Fish (1998) [5]; and Fish et al. (2014) [18]. *This figure is based on the figure by Fish et al. (2014) [18].

The drag coefficients calculated in the present study are compared with theoretical values for a flat plate and previous data for dolphins or dolphin models summarized by Fish (2014) [18] in Fig 13. The friction drag coefficient (*C*_*D*, friction_) determined by our calculations grossly agrees with the theoretical friction drag coefficient for a flat plate with a turbulent boundary layer (0.072 × *Re*^-0.2^). In other words, the pressure drag component (*C*_*D*, pressure_) appears to be the main source of the difference between our *C*_*D*_ and the theoretical friction drag coefficient for a flat plate with a turbulent boundary layer. The values that we computed for *C*_*D*_ are within a range of the previously reported values for dolphins or dolphin models (black, blue, red, and green plots in Fig 13). For earlier CFD simulations of dolphins, Riedeberger and Rist (2012) conducted a CFD simulation of a common dolphin (*Delphinus delphis*) using a realistic 3-D model; the computed value of *C*_*D*_ was 4 × 10^−3^ for *Re* in the range of 5.5 × 10^5^ to 1 × 10^7^ [20]. Our *C*_*D*_ values of 6.1 × 10^−3^ to 4.7 × 10^−3^ for *Re* equal to 1 × 10^6^ to 1 × 10^7^ do not differ significantly from the results by Riedeberger et al., despite the that different species that were used.

Our CFD calculation using a realistic 3-D model also suggested that pressure drag contributes considerably to the total drag even at zero angle of attack, as indicated in Table 1. Note that it is expected that the drag coefficients increase when the angle of attack increases since our model is rigid and straight. Moreover, unsteady body bending like a real dolphin in active propulsion may lead to an increase in the drag coefficients as explained in the Methods section.

### Asymmetry in acceleration and thrust generation between the downstroke and upstroke

The most notable finding in this study was that the dolphin greatly accelerated with downstrokes but accelerated very little with upstrokes during short-duration acceleration to reach more than 8 m s^-1^ (the 1st to 8th strokes in Fig 7(C)). Based on the value of *C*_*D*_ calculated by the CFD simulation, the resultant stroke-averaged thrust values were greater during downstrokes than during upstrokes (Fig 11(A)). Videler and Kamermans (1985) reported a similar asymmetry for slowly cruising dolphins: their bottlenose dolphin (*Tursiops truncatus*) and Guiana dolphin (*Sotalia guianensis*) always accelerated during downstrokes and decelerated during upstrokes when they swam at speeds of 1.77 to 3.17 m s^-1^[17]. They also expected that at high speeds the upstrokes might increase its share in the thrust and will be at least high enough to balance the drag. Our result of the acceleration measurement at higher speed ranging from 5.0 to 8.8 m s^-1^ (the 1st to 8th strokes), however, did not support their expectation but show that the share of the upstroke in thrust was still small and the thrust by the upstroke could be less than the estimated drag.

Even though the net thrust (i.e. resultant force of the fluke’s thrust and the other parts’ drag, that is, the total force minus buoyancy and gravity forces in Eq (6)) during upstroke was smaller than during downstroke in the rapid acceleration stage until the 8th stroke, it still generated considerable thrust (i.e. *T* in Eq (6)) except for the 6th stroke. Hence, taking into consideration that our estimation on drag should be conservative and undulating dolphin might be subject to greater drag, our observation suggests that at high-speed swimming the dolphin could generate thrust during both downstroke and upstroke, while downstroke appeared to generate more thrust than upstroke. On the other hand, during the final beat before the leap after the acceleration at 9.4 m s^-1^ (the 13th and 14th stroke), even the downstroke did not accelerate the dolphin (Fig 7(C)) suggesting that the net thrust contribution of downstrokes and upstrokes depended on both speed and acceleration conditions.

Note that the position of the tracking marker relative to the center of mass affects the results of velocity and acceleration. In Videler and Kemermans’s study, the marker was located at the dorsal ridge behind the dorsal fin. Our preliminary analysis, however, revealed that the acceleration values based on the dorsal marker (Fig 1(A)) were emphasized, resulting in greater magnitude of acceleration during both downstroke and upstroke than those based on the lateral markers. This difference was assumed to be due to longitudinal pitching and bending of the dolphin. Lang and Daybell also reported that time-series data of velocity at a dorsal part fluctuated more than those at a beak part [7]. Thus, the lateral markers were chosen in the present study since they were expected to be closer to the center of mass in the lateral view than the dorsal marker. Nevertheless, asymmetry in thrust generation between the downstroke and upstroke was observed.

Fish et al. (2014) visualized the flow field of a wake behind the fluke of bottlenose dolphins (*Tursiops truncatus*) with DPIV using a bubble sheet for slow-speed swimming up to 3.7 m s^-1^ [18]. The published video [28] shows that a strong vortex is shed from the fluke at the start of an upstroke, which implies generation of strong hydrodynamic force during the preceding downstroke. However, vortex shedding after the upstroke was not confirmed since the fluke moved out of the field of view of the video. Instead, the authors commented that a pair of counter-rotating vortices was generated for each fluke beat (i.e. downstroke and upstroke, or upstroke and downstroke) in the paper. Their computational analysis based on observed wake circulation indicated that the fluke generated hydrodynamic force during each stroke. The direction of the calculated force, however, was not clarified.

In summary, during the rapidly accelerating swimming in our observation, the dolphin generated positive net thrust predominantly during each downstroke of its fluke and recover during the upstroke with less or negative net thrust. The estimation of thrust (i.e. *T* in Eq (6)) depends on the estimation of the drag. Since our estimation of *C*_*D*_ was conservative due to the static CFD simulation, the calculated thrust was consequently conservative. For further investigation of the difference in thrust generation between the downstroke and upstroke, further unsteady CFD simulations or fluid dynamic experiments with a moving model are desired.

### Power-to-mass ratios during high-speed burst swimming

The calculated power-to-mass ratio in the present study was compared with previous data for dolphins and orca whales when swimming faster than 5 m s^-1^, as presented in Table 3. The table was constructed based on the table by Fish et al. (2014) [18]: slow-speed cases less than 5 m s^-1^ were removed, a tail-standing case by Isogai (2014) [29] was added, and the maximum fluke-beat-averaged and stroke-averaged values in the present study were added.

**Table 3:** Thrust power of dolphins in high-speed swimming and standing swimming.

Species	Mass (kg)	Length (m)	Velocity (m s^-1^)	Method*	Thrust power (W)	Power-to-weight ratio (W kg^-1^)	Source
*Delphinus delphis*	90.7	1.8	10.1	D	1938.8	21.4	Gray (1936) [1]
*Phocoena phocoena*	24.0	1.2	7.6	D	447.4	18.6	Gray (1936) [1]
*Orcinus orca*	1645.4	4.8	8.0	US	36259.6	22.0	Fish (1998) [5]
*Pseudorca crassidens*	535.8	3.8	7.5	US	12065.7	22.5	Fish (1998) [5]
*Stenella attenuata*	52.7	1.86	11.05	A	4517.8	85.7	Lang and Pryor (1966) [15]
*Tursiops truncatus*	214.9	2.6	6.0	US	5090.9	23.7	Fish (1998) [5]
*Tursiops truncatus*	138	2.3	Standing	NS	8582 (fluke-beat-averaged), 18395 (instantaneous)	62.2 (fluke-beat-averaged), 133.3 (instantaneous)	Isogai (2014) [29]
*Lagenorhynchus obliquidens*	91.0	2.1	5.5	QS	6180	67.9	Webb (1975) [11]
*Lagenorhynchus obliquidens*	91.0	2.1	5.5	US	4030	44.3	Webb (1975) [11]
*Lagenorhynchus obliquidens*	91.0	2.1	5.2	US	1223.7	13.4	Yates (1983) [12]
*Lagenorhynchus obliquidens*	110	2.1	7.4 (No. 3, 4)	A	5.3 × 10^3^ (fluke-beat-averaged)	48	Present study
*Lagenorhynchus obliquidens*	110	2.1	8.4 (No. 5)	A	9.9 × 10^3^ (stroke-averaged)	90	Present study

*Method: A, acceleration (Lang and Daybell (1963) [7]); D, drag-based (Gray (1936) [1]); QS, quasi-steady (Parry (1949) [9]); US, unsteady lifting surface (Lighthill (1969) [30], Chopra and Kambe (1977) [4]); NS, unsteady numerical simulation of Navier-Stokes equations.

**This table is based on the table by Fish et al. (2014) [18].

The dolphin’s size, mass, and velocity in the present study were similar to that in the original study by Gray (1936) for *Delphinus delphis*. The power-to-mass ratio of *Delphinus delphis* computed by Gray assuming a turbulent boundary layer was 21.4 W kg^-1^, which corresponds to only 45% of the 48 W/kg determined in our study. Since Gray considered only frictional drag and neglected pressure drag in his estimate of fluid drag, his estimate of the fluid drag and the resultant thrust would underestimate the actual drag by 30% to 40% according to our CFD calculation (Table 2).

Lang and Pryor (1966) [15] attempted to measure maximum speeds and accelerations of *Stenella attenuata* on a 25-m straight course in a 35 m by 300 m lagoon with a depth of 4 m. The maximum speed was recorded as 11.05 m s^-1^ after 2 s of acceleration, and the maximum power was observed 0.5 s before reaching the top speed. The reported peak power-to-mass ratio was 85.7 W kg^-1^, which was close to our maximum stroke-averaged value of 90 W kg^-1^, although Lang and Pryor’s paper does not specify whether the calculated power corresponded to the fluke-beat-averaged value, the stroke-averaged value, the frame-by-frame value, or another metric.

Previously reported power-to-mass ratios for *Lagenorhynchus obliquidens* vary with the method of calculation: 67.9 W kg^-1^ using a quasi-steady model by Webb (1975) [11], 44.3 W kg^-1^ using an unsteady lifting surface theory by Webb (1975) [11], and 13.4 W kg^-1^ using another unsteady lifting surface theory by Yates (1983) [12]. The maximum fluke-beat-averaged value in our case (48 W kg^-1^) is within the above range, whereas the maximum stroke-averaged value (90 W kg^-1^) exceeded the previous range. In general, the calculation of the thrust of a beating fluke using fluid dynamic theory tends to be sensitive to the angle of attack of the fluke and wing characteristics (i.e., the lift and drag curves of the fluke profile). In addition, the effect of unsteady vortices caused by a high angle of attack and elastic deformation of the fluke is challenging to incorporate. Fish and Rohr (1999) noted that the quasi-steady model appears to yield higher values of thrust power than the other models [13]. Furthermore, methods based on the equations of motion of the body, as in the present study and that of Lang and Pryor (1966), depend on the accuracy of the calculated acceleration and the estimated drag coefficient used in the equations. Instantaneous values for the acceleration tend to fluctuate greatly, as shown in Fig 7(C), since the acceleration is calculated as a second-order discrete differential of the position with a small time step. Low-pass filtering was used in the present study to compute the fluke-beat-averaged and stroke-averaged values to suppress high-frequency fluctuations and provide reliable results.

The maximum stroke-averaged power-to-mass ratio of 90 W kg^-1^ in the present study is high relative to previous data except for that of Isogai (2014), who reported a comparable value of 133.3 W kg^-1^ for *Tursiops truncatus* in short-duration tail standing [29], in which the dolphin’s body maintained an upright position in the air while only the fluke was beating underwater. In this tail-standing mode of swimming, the dolphin is expected to generate a large amount of power to support its weight without buoyancy; there is no need to consider fluid drag acting on the body in the calculation of the thrust power. Isogai created a time-varying 3-D model of the fluke that considered elastic deformation and calculated the thrust force using an unsteady CFD simulation, providing a time-varying curve for the thrust power. According to Isogai’s study, the dolphin generated almost symmetrical positive thrust in both the downstroke and upstroke, and the instantaneous power-to-mass ratio was 133.3 W kg^-1^, which is 2.14 times greater than that of the fluke-beat-averaged value of 62.2 W kg^-1^. Similarly, our maximum stroke-averaged value of 90 W kg^-1^ is 1.9 times larger than the maximum fluke-beat-averaged value of 48 W kg^-1^. These results suggest that the instantaneous or stroke-averaged power-to-mass ratio of dolphins could be considerably greater than the conventional fluke-beat-averaged values. Note that our estimation of *C*_*D*_ was conservative due to the steady CFD simulation with the static model, hence our estimation of the thrust and the thrust power-to-mass ratio was also conservative, and the factual thrust and thrust power-to-mass ratio may be even stronger. In future research, an undulating body model is demanded to replace the rigid body model, then the estimation on drag, thrust and thrust power may become more accurate, which may further expand our understanding of the swimming ability of dolphins and the mechanism of dolphin swimming.

### Biomimetics applications

From the viewpoint of biomimetic engineering, dolphins are perhaps superior to submarine-type underwater vehicles generating thrust with conventional rotational propellers in terms of quick acceleration performance owing to their intense beating of a fluke. In the development of biomimetic underwater robots [31–36], not only statistically averaged kinematics data of model animals but also detailed time-series data regarding a specific individual with known morphology are informative to investigate the mechanics and dynamics of locomotion. This paper provides, to our knowledge, the first time-series kinematics data of a specific dolphin with a detailed morphology in a specific burst-acceleration swimming with successive fluke beats, which the developers of biomimetic underwater robots could refer to. Moreover, the estimated thrust power-to-mass ratio of the dolphin could be used for comparison of propulsion performances with those biomimetic robots. The asymmetry in acceleration and thrust generation between the downstroke and upstroke of dolphin swimming especially deserves further investigation, as a potential source of reference for design of biomimetic swimming robots propelled by tail beating.

## Ethics

The experimental procedure was approved by Yokohama Hakkeijima Sea Paradise, the National Museum of Nature and Science, and Animal Experiment Committee of Chiba University. No dolphin was harmed during any part of the study. The animal welfare and housing condition details of the dolphin are provided in S1 Appendix.

## Supporting information

S1 Appendix Dolphin’s welfare and housing conditions. (PDF)

S1 Movie Burst accelerating swimming of a Pacific white-sided dolphin (*Lagenorhynchus obliquidens*) toward a vertical leap. (MP4)

S2 Movie Trajectories of the lateral markers. (MP4)

S1 File Original model of a frozen dolphin (*Lagenorhynchus obliquidens*). Number of meshes was reduced to save the file size.
(STL)

S2 File Reconstructed symmetrical model of the dolphin. Number of meshes was reduced to save the file size.
(STL)

S1 Fig Color contour of *y*^+^ at the first prism layer. (PDF)

S1 Dataset Reconstructed 3-D coordinates of the beak, right lateral, left lateral, dorsal, base of the fluke, median notch of the fluke, left tip of the fluke, and right tip of the fluke markers. (XLSX)

S2 Dataset Lowpass-filtered 3-D coordinates of the beak, right lateral, left lateral, dorsal, base of the fluke, median notch of the fluke, left tip of the fluke, and right tip of the fluke markers. (XLSX)

S3 Dataset Timings of key events. (XLSX)

S4 Dataset Time-series data of velocity and acceleration. (XLSX)

S5 Dataset Time-series data of force and drag coefficient. (XLSX)

S6 Dataset Stroke-averaged and flukebeat-averaged velocity, acceleration, force, and power. (XLSX)
