# Supplementary material for: Measurement of time-varying kinematics of a dolphin in burst accelerating swimming
Source: PLoS One. 2019 Jan 30;14(1):e0210860. doi: 10.1371/journal.pone.0210860 (PMC6353170; doi:10.1371/journal.pone.0210860)
Supplement: S1 Appendix — (PDF) [file pone.0210860.s001.pdf]

S1 Appendix. The animal welfare and housing condition details of the dolphins at Yokohama Hakkeijima Sea Paradise

1. Housing and feeding conditions

Yokohama Hakkeijima Sea Paradise raised nine Pacific white-sided dolphins (*Lagenorhynchus obliquidens*) including the dolphin we measured and a single bottlenose dolphin (*Tursiops truncatus*) at that time. There are three water tanks for the dolphins as summarized in Table S1-1. The two research tanks are connected to the exhibition tank. The five dolphins were raised in the northern research tank, and the other five dolphins were raised in the southern research tank.

The dolphins were fed seven times per day: four times after exhibitions and three times after practices. Amount of each feeding was approximately 1 kg (i.e. 7 kg per day) for each dolphin. Main feed was horse mackerels.

Table S1-1. Water tanks for the dolphins at Yokohama Hakkeijima Sea Paradise

Water tank	Approximate size and water depth	Water temperature	Main usage
Northern research tank	10 m × 6.2 m × 3 m	22°C	Raising the five Pacific white-sided dolphins
Southern research tank	15.7 m × 10 m × 3 m	22°C	Raising the four Pacific white-sided dolphins and the bottlenose dolphin
Exhibition tank	35 m × 25 m × 6 m	23°C	Exhibition and recreation for the dolphins

2. Environmental enrichment

Each exhibition and practice in the exhibition tank lasts 5 minutes and 10 minutes, respectively. The dolphins participate in the exhibitions and practices three times and four times per day, respectively. The remaining time other than the exhibitions and practices was free time. During the free time, the dolphins can play in various ways. For example, the aquarium staffs hose the dolphins from the ground and swim together in the tanks. The dolphins also can play by themselves with floating buoys or ice blocks which are provided by the staffs properly.
