# Supplementary figures and images for: Measurement of time-varying kinematics of a dolphin in burst accelerating swimming
Source: PLoS One. 2019 Jan 30;14(1):e0210860. doi: 10.1371/journal.pone.0210860 (PMC6353170; doi:10.1371/journal.pone.0210860)

**(A)  $Re = 1 \times 10^6$**

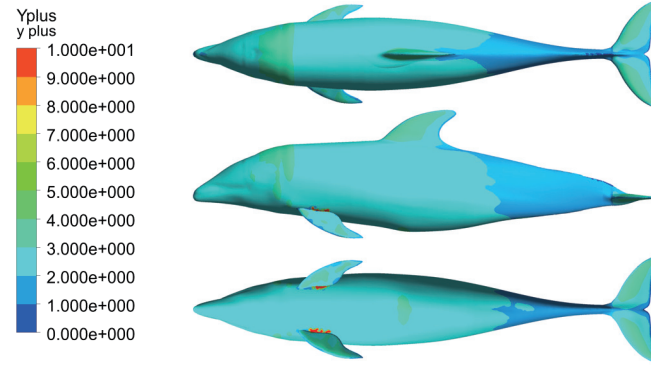

**(B)  $Re = 1 \times 10^7$**

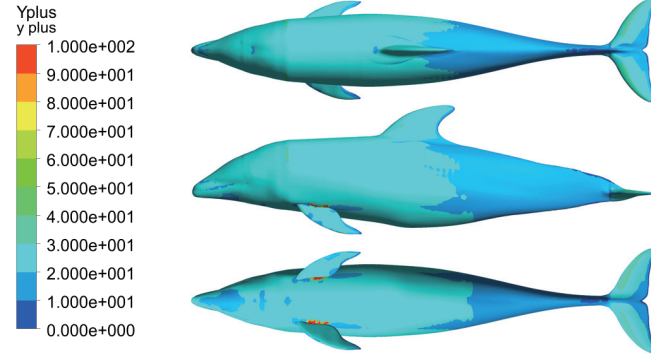

**(C)  $Re = 2 \times 10^7$**

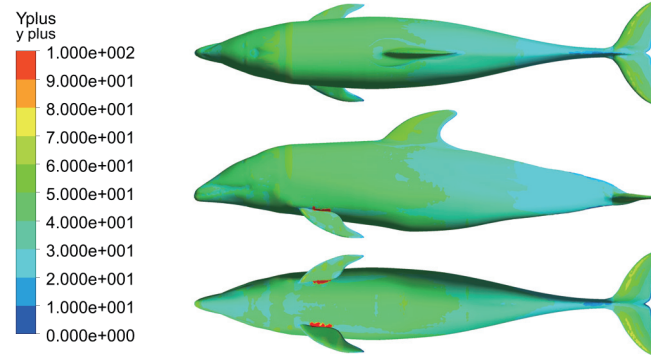

**(D)  $Re = 3 \times 10^7$**

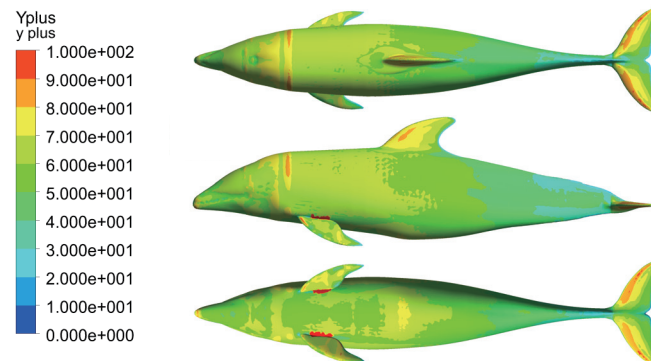

**(E)  $Re = 4 \times 10^7$**

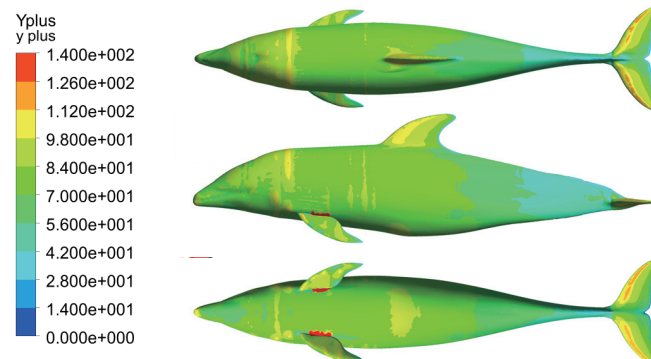

Supplement: S1 Fig — (PDF) [file pone.0210860.s006.pdf]
